# Strain in Halide Perovskites and Solar Cell Stability: Accelerated Stress Tests under Bias Voltage

**DOI:** 10.1021/acsenergylett.4c02822

**Published:** 2024-12-26

**Authors:** Fanny Baumann, Masoud Karimipour, Jessica Padilla-Pantoja, Emigdio Chávez-Angel, Jose Manuel Caicedo Roque, Rémy Pouteaux, Alex Alcalá Ibarra, Sonia R. Raga, José Santiso, Monica Lira-Cantu

**Affiliations:** Catalan Institute of Nanoscience and Nanotechnology (ICN2), CSIC and the Barcelona Institute of Science and Technology (BIST), Building ICN2, Campus UAB, E-08193 Bellaterra, Barcelona, Spain

## Abstract

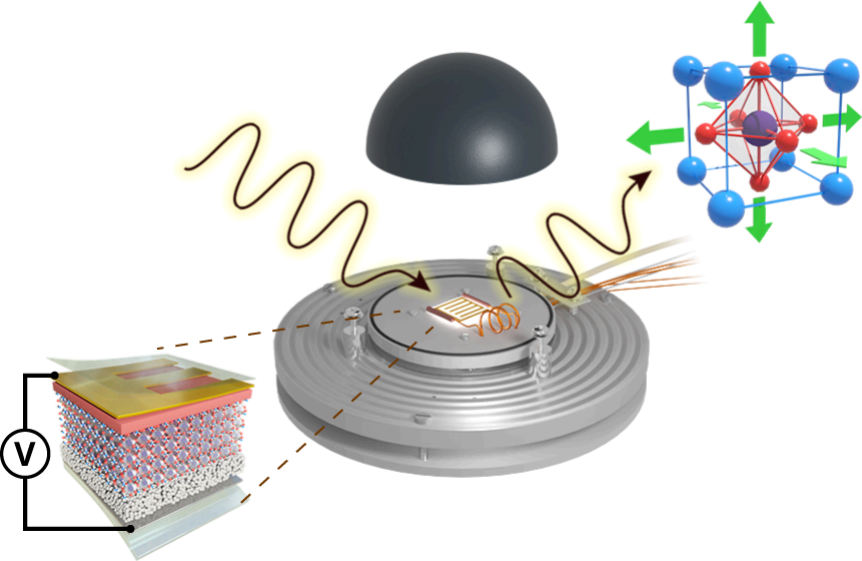

The lifespan of halide perovskite solar cells (PSCs) is currently a major concern for the implementation and commercialization of the technology. Tensile and compressive strain alters the halide perovskite (HP) lattice under operando conditions, affecting PSC stability. However, the mechanisms governing strain responses are still unknown. In this work, we monitored the evolution of strain in PSCs during accelerated stability tests under continuous light irradiation and bias-voltage. Additive engineering led to a compression of the HP lattice, improving PSC stability. The temporal evolution of the HP lattice, together with the electrical response of devices employing modified and reference HP, was tracked by *in situ* X-ray diffraction, photoluminescence, and quasi *in situ* electrochemical impedance spectroscopy. Our results demonstrate a good correlation between lattice expansion (strain) and the device’s current decay and stability. Additionally, and contrary to the current understanding, we observed that lattice compression in HP was not sufficient to ensure protection against degradation.

Although perovskite solar cell (PSC) technology has demonstrated record efficiencies exceeding 26%, enhancing device stability and lifespan remain critical factors for its successful commercialization. Among many efforts, *strain engineering* has become an effective method to enhance the PSC performance and stability. Strain in halide perovskites (HPs) is the deviation from the isolated most favored lattice configuration/symmetry, in response to applied intrinsic or extrinsic stimuli. For example, it can be present as elastic strain, including deformation of the lattice from pressure, or chemical strain, including stretching of bonds to accompany, e.g., heterogeneous crystallization and chemical composition changes. Strain is common in all structures having a mixed composition or an interface. Due to the soft nature of HPs, strain can occur jointly in two opposite directions (e.g., Poisson effect, where in-plane tension leads to out-of-plane compression and vice versa) or in an isotropic fashion (e.g., expansion/compression) and in a gradual/local fashion (microstrain) from, e.g., a strained surface into the bulk of a crystal or more generally in the material (macrostrain).^1^*Tensile strain* is known to weaken Pb–I bonds and A-site cation interaction with the inorganic framework, while *compressive strain* can narrow the bandgap and increase ion migration activation energy.^2^ Variation in strain can impact ion migration, optoelectronic properties (bandgap, defect density, carrier dynamics), phase transition activation energy, and interface properties. Therefore, modulation of strain is crucial to reduce interface delamination and material decomposition, minimize the formation of undesirable defects during fabrication, reduce nonradiative recombination, and ultimately improve PSC efficiency and stability. Various approaches have been employed to modulate strain and achieve highly stable PSCs, including tuning the HP precursor composition (A, B or X-site manipulation);^3^ controlling thin film formation (crystallization additives, annealing temperature); additive engineering of the HP; and interface strain modulation.^4^ Organic additives have been employed to modify strain in the HP film, e.g.: 2,2- diamino-[1,1-biphenyl]-4,4-dicarboxylic acid (DBDA), adenosine triphosphate (ATP), p-toluene sulfonate (p-TS),^5^ trioctylphosphine oxide (TOPO),^6^ and the ionic imidazolium tetrafluoroborate (IM^+^BF_4_^–^).^7^ It was observed that the relaxation of strain, in all cases, resulted in improved PSC stability.^8,9^ In general, the impact of lattice compression in the HP has been observed as enhanced PSC stability, while lattice expansion has had the opposite effect, observed as reduced solar cell lifetime. Despite significant progress where existing studies relate the initial or final state of the HP strain to PSC stability, monitoring the evolution of strain throughout the complete stability analysis has been overlooked. Hence, the mechanism of strain evolution and the relation between lattice strain progression and device stability during *in situ* and *operando* conditions are still unexplored, even less so under accelerated tests.

## Monitoring Strain via In Situ Characterization

**Figure 1:**
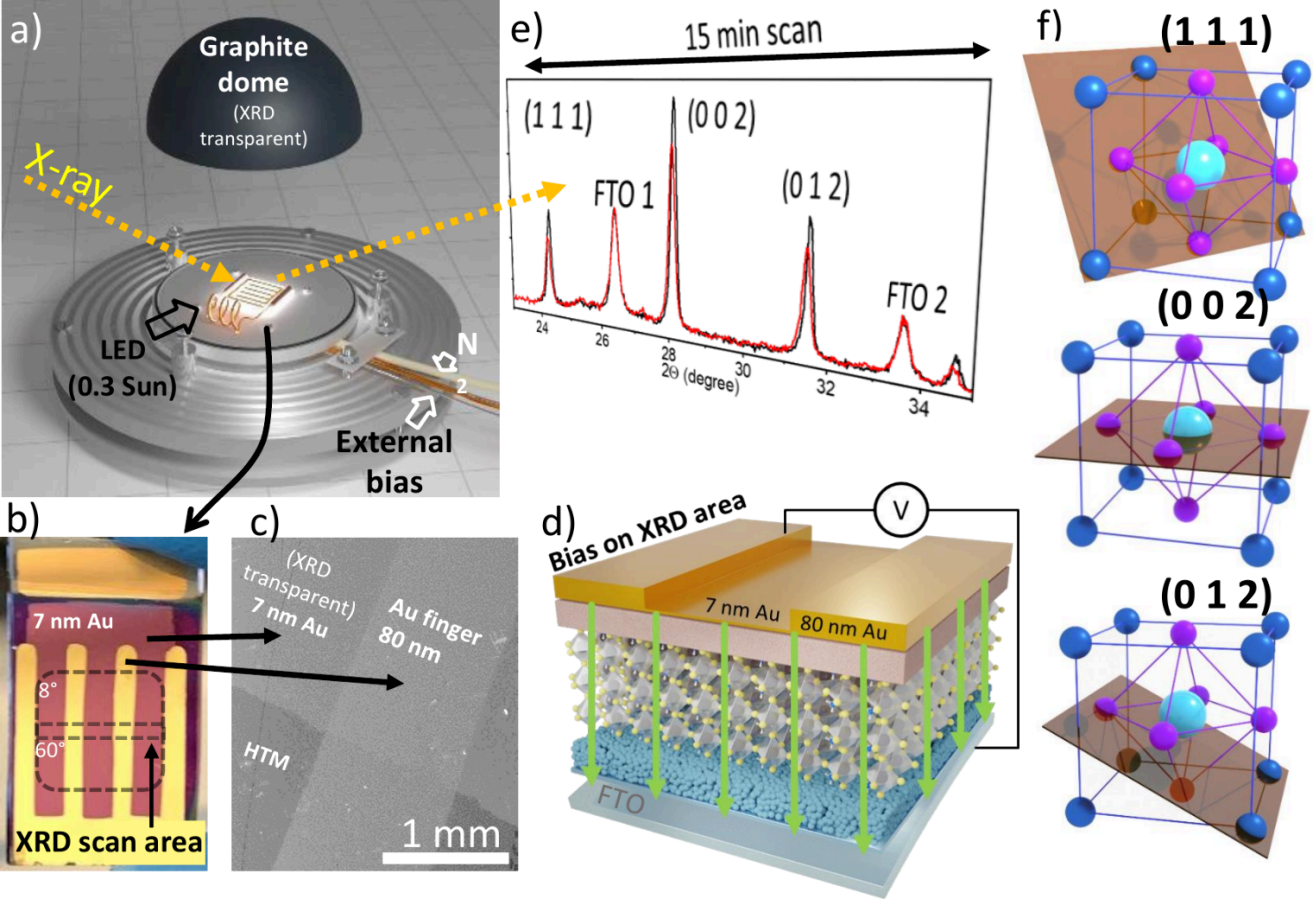
(a) Schematic of the XRD-stage sample-holder, including a graphite dome, N_2_ inlet, and illumination. (b) Picture of the metal electrode with 80 nm thick Au fingers and semitransparent 7 nm thin Au film covering the entire XRD-scanned area. Dashed lines mark the 2θ-dependent XRD scan area. (c) SEM image of intersecting Au layers on bare HTM. (d) Schematic of the PSC, with green arrows indicating the electric field distribution, marked Au and FTO electrodes. (e) Region of the scanned XRD during in situ bias stress (2θ = 23.8° to 35.4°, ∼15 min). (f) Representation of perovskite crystalline planes (111), (002) and (012).

X-ray diffraction (XRD) is one of the most common techniques to characterize bulk average lattice constants (out-of-plane macrostrain) of the HP in a PSC. However, a key challenge is encountered during *in situ* XRD analysis of biased solar cells: the attenuation of X-rays by strong interference with the standard 80 nm-thick metal electrode, preventing X-rays from the perovskite (the diffraction pattern) from reaching the detector. Existing reports on *in situ* XRD with electrically connected PSC have mainly focused on degradation caused by temperature, illumination and/or moisture, where the perovskite XRD pattern is obtained primarily from the nonbiased area outside the metal electrode.^10−13^ Unless the experiment uses high-energy synchrotron beams, the recorded XRD signal from HP beneath or outside the active area may be substantially different. Nevertheless, high-intensity synchrotron radiation could also damage the perovskite. Long degradation studies on statistically important numbers of samples could be prohibitively costly for most research groups, and seeking an alternative approach to use readily available XRD systems for “common lab *in-situ* testing” would be a beneficial step in the right direction to encourage frequent *in situ* studies in line with stability evaluation. Inspired by pioneer work by Kim et al.,^14^ we developed an electrode consisting of 80 nm thick Au fingers (thermally evaporated) with a top 7 nm thin Au layer (electron beam evaporated), filling the active area around and between the thicker fingers (Figure 1b–d). This electrode design allowed for bias distribution throughout the XRD probe area (∼9 × 9 mm^2^ at 2θ = 8° to ∼9 × 1.5 mm^2^ at 2θ = 60°, Figures 1b and S1).

To explore structural changes induced by light and electric bias and their relation to stability, we developed a sample holder configuration specialized for performing *in situ* (reflective parallel beam) XRD on a PSC with our customized electrode for bias application. The setup incorporated electrical connections, a cold white LED, and a protective N_2_ flow enclosed by a graphite dome (Figures 1a and S2). Our homemade setup and custom-designed electrodes enabled simultaneous XRD analysis on a fully operational PSC working under N_2_ at 0.3 sun, while applying bias voltage and monitoring the electrical response. To improve the frequency of XRD monitoring, we narrowed the 2θ acquisition range to 23.8°–35.4°, capturing three characteristic diffraction peaks of HP planes, (111), (002) and (012) (Figure 1e,f), and two fluoride-doped tin oxide (FTO) peaks corresponding to the transparent bottom electrode on the glass substrate (Figure 1e,d). The FTO (110) peak (JCDPS card No. [46-1088]) at 2θ ≈ 26.4° was used as control, allowing us to correct for possible misalignments during experiments and between samples since no differences are expected from the identical substrate during analyses. By the shortened scan, the acquisition time of each XRD was reduced to ∼15 min.

## Lattice Compression via Additive Engineering

**Figure 2:**
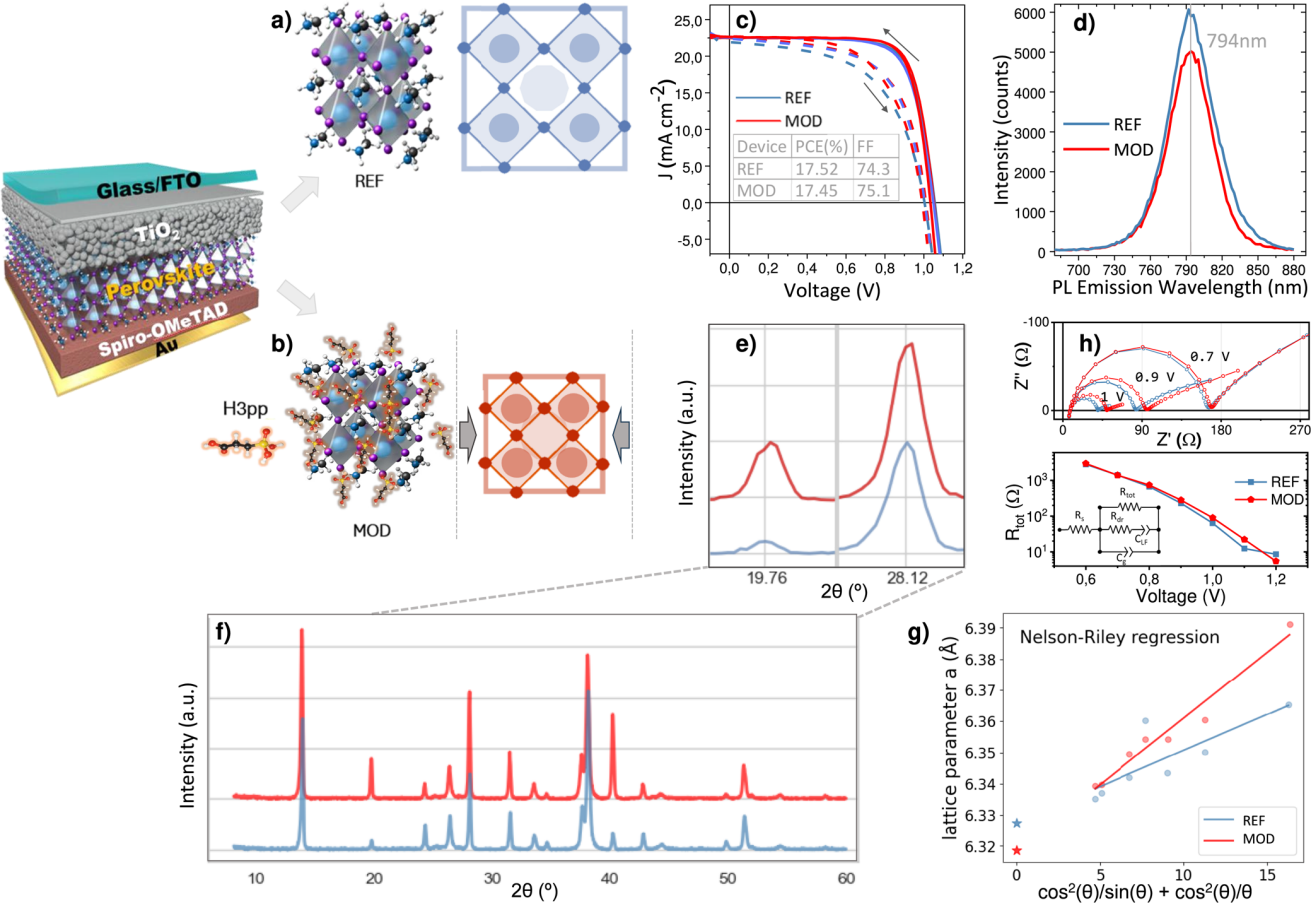
With (MOD) and without (REF) additive engineering of the halide perovskite by employing the H3pp organic molecular additive in the absorber precursor. (a,b) Schematic representation of the compressed (MOD) and uncompressed (REF) lattice. (c) JV curves of PSC from 4 test samples of the experimental batch. (d) PL spectra. (e,f) X-ray diffraction (XRD) of devices before experiments. (g) Nelson-Riley regression of a-values (before any alignment). (h) EIS Nyquist and fitted R_rec_ plots using the equivalent circuit in the inset.

**Figure 3:**
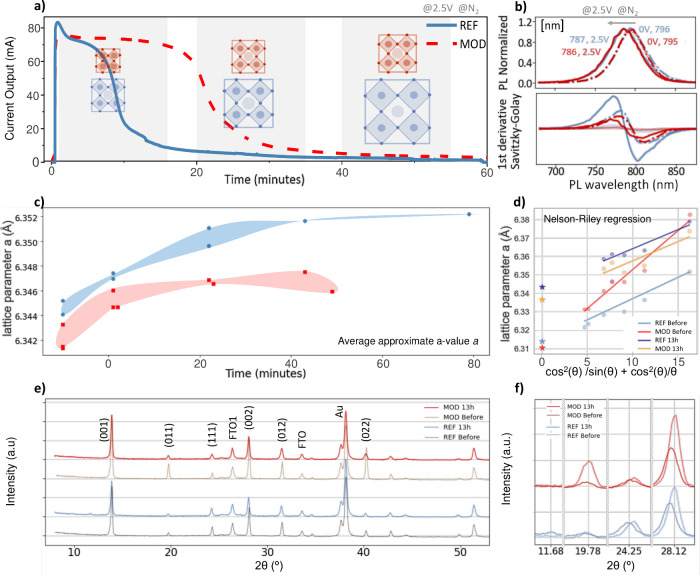
Lattice expansion observed through the accelerated stability analysis of PSC with (MOD) and without (REF) additive engineering. (a) Current decay observed over time during the stability test under bias voltage and illumination. We include a schematic representation of the strain evolution observed for the REF and MOD samples, assumed cubic. REF and MOD: (b) PL shift and corresponding EL and quenched PL when applying bias. (c) Average a-value change as calculated by Braggs law from the perovskite XRD peaks center found by Pseudo-Voigt. (d) Nelson-Riley regression analysis of a-values (after alignment) from the scans in (e) XRD scans of PSC before and PSC at 13 h of ongoing bias. (f) Zoom in on XRD peaks in part e: secondary phase peak at 2θ = 11.68°, and perovskite peaks (011), (111) and (002).

To study the effect of lattice compression on PSC stability enhancement, we employed additive engineering methodology to a control HP sample. PSCs with the H3pp additive, hereafter indicated as “modified” (MOD), have been shown to exhibit significantly enhanced long-term stability^15^ but give very similar optoelectronic properties to control devices, hereafter indicated as “control or reference” (REF). The molecules do not reside inside the crystal (as has been shown by nuclear magnetic resonance studies) but locate predominately at the grain boundaries, and the additive has been demonstrated to predominantly passivate shallow defects in HP via interaction with iodine vacancies and the A-site cation.^15^ Thus, a quadruple cation HP precursor solution, with the composition Rb_0.05_Cs_0.05_MA_0.15_FA_0.75_Pb_1.05_(I_0.95_Br_0.05_)_3_, was modified with the organic additive 3-phosphonopropionic acid (H3pp). A lattice compression was detected via evaluation by XRD of identical samples with (MOD) and without (REF) the H3pp additive (Figures 2f and S3). Figure 2a–b shows a schematic representation of the lattice compression observed for the HP lattice after the addition of the organic H3pp additive. The as-prepared PSCs revealed differences in the diffraction peaks corresponding to the HP crystallographic planes. Consistently, the MOD peaks were shifted +0.02° 2θ compared to REF peaks, while the FTO peaks remained unaltered (Figure S3.1). Further analysis by Nelson-Riley regression showed that a small lattice compression of the HP was induced by H3pp additive engineering within identical samples (Figures 2g, 3d, and S3.2–3.3). Additionally, MOD HP showed a higher peak intensity at 2θ ≈ 19.78° (011) in comparison with the REF sample (Figures 2e–f and S3.4d), indicating a change in the preferential crystal orientation when employing H3pp. Current density–voltage (*J–V*) curves, photoluminescence (PL) spectroscopy and electrochemical impedance spectroscopy (EIS) at various applied voltages, 0.7–1.2 V, showed that MOD and REF devices showcased similar photovoltaic performance (Figure 2c), bandgap (Figure 2d), and recombination resistance (*R*_*rec*_) (Figure 2g), in good agreement with previous works.^15,16^ In addition, the characteristic times of the low frequency EIS signal, related to the ionic conductivity in the HP (Figure S4a),^17,18^ showed similar results with and without H3pp. This suggests that the small compression in MOD HP had a minimal influence on ion dynamics, initially. Any residual strain from fabrication was not transferred to the HP layer (Figure S5).

## Lattice Expansion during Accelerated Stress Tests

To further analyze the effect of lattice compression observed in the as-prepared MOD samples on the long-term structural stability of the HP, we ran accelerated stability tests by applying 1.2 and 2.5 V forward bias voltages, under continuous illumination conditions, leading to high excess carrier conditions in the HP. All measurements were carried out at room temperature, with inert N_2_ flow and using an LED illumination source.^19,20^Figure 3a depicts the output current (mA) response during accelerated testing for a representative REF and MOD PSC at 2.5 V bias and 0.3 sun. In general, the current in all of the analyzed devices showed an initial increase, followed by a brief stabilization, before fast decay as the PSC degraded. The reproducibility on several other PSCs is shown in Figures S6 and S8. Simultaneously with the current evolution, we observed that all HP XRD peaks shifted to lower angles and decreased in intensity (Figure 3c–f and Figure S3.4). The reduction of peak intensity suggests a loss of crystallinity or decomposition; however, the relative microstructure was preserved (Figure S3.4c–d). This change was dependent on bias and independent of only illumination or X-ray exposure (Figures S7 and S8), and without involvement of other visible features (Figure S9). Both current and XRD changes were more pronounced during the first 30 min of measurements (Figures 3a,c and S6), suggesting a common mechanism behind electrical and structural changes. To visualize the rate of the shift, each perovskite peak was fitted with a pseudo-Voigt function to calculate the interplanar out-of-plane distance *d* for each Bragg reflection as per Bragg’s law^21^ and the corresponding lattice parameter *a* (Figure S3). The REF PSCs exhibited earlier current decays in comparison to the MOD PSCs, consistent with superior stability post H3pp modification.^15,16^ The *a*-value progressions of REF and MOD are shown in Figure 3c. During testing, REF HP exhibited a gradual XRD shift that ultimately reached up to above Δ2θ = −0.04° and a shift in the out-of-plane lattice parameter Δ*a* ≈ +0.02 Å (Figures 3d and S3.4). MOD HPs showed a smaller shift of Δ2θ ≈ −0.02° within the first 20 min of testing, with Δ*a* ≈ +0.01 Å, reaching marginally above the initial value of REF. Notably, beyond this point, the shift rate of the MOD HP diffraction peaks reduced significantly, and MOD HP did not expand much further. After prolonged bias, PSCs were prone to short-circuits, with the observation that MOD PSCs seemed to short-circuit once passing approximate *a*-values of over 6.347 Å. Reference PSCs did also short-circuit, but this response was observed after larger changes.

In order to detect the appearance of any XRD peaks related to secondary phases produced from HP decomposition (expected at the end of the accelerated tests), long XRD scans from 2θ = 8° to 60°, of 60 min duration, were recorded before and at the end of the bias tests (13 h, Figure 3e). These revealed no formation of impurities related to hydration, and a preservation of the cubic structure of the remaining perovskite phase (Figure 3d). After prolonged bias and illumination, the REF PSC showed a hint of a peak at 11.6° 2θ (Figure 3e–f), assigned to the delta or “yellow-phase” (see section S10).^22,23^

In summary, upon applying an elevated bias of 2.5 V to PCE operating under illumination, we observed that, despite the lattice compression provoked in the MOD samples before stability analysis, the HP lattice of both REF and MOD samples continuously expanded with time. In the case of the additive-engineered perovskite with the H3pp organic molecule (MOD), the HP was more resistant to lattice expansion, and the observed changes leveled out earlier than for the REF HP. Additionally, the MOD HP lattice, which initiated in a more compressed state, remained in a relatively more compressed state at any given time during the bias and illumination test compared with REF HP. Nevertheless, we undoubtedly observed lattice expansion in both perovskite samples during the stability tests, independent of their initial lattice state. Our results indicate that lattice compression had a positive effect on HP stability over time as reported.^4,8,9,24−26^ However, our results also suggest that ensuring initial lattice compression in the HP is not enough to guarantee long-term stability, since both lattice expansion and current decay in the additive-engineered sample were still observed with time.

## Phenomenological Effects Observed via In Situ PL and Quasi In Situ EIS

To better understand what phenomenological effects are present during accelerated stability tests, we carried out *in situ* PL on PSCs with (MOD) and without (REF) the organic additive H3pp. Similarly, we monitored the current output obtained at an applied bias voltage simultaneously as the PL was collected during pulsed excitation by a 453.6 nm laser from the glass side of the cell. A clear and reproducible PL response was detected during the accelerated test, and the ramification of the change depended on the magnitude of the bias voltage (Figure S8). The application of 2.5 V bias promptly displaced the PL center toward wavelengths below 790 nm (Figure 3b). A blueshift from, e.g., ∼796 to ∼791 nm in the PL wavelength corresponds to a shift in the bandgap of approximately 10 meV, and using this relation, the observed PL change represented a bandgap shift from ∼1.56 eV to above 1.59 eV (mean change ∼3.5%), a shift reasonably attributed to the magnitude of expansive strain observed in XRD. In parallel, the intensity of PL decreased by 2 orders of magnitude in less than 5 min (Figure S11). The response was similar for REF and MOD devices and in agreement with a lattice expansion and loss of crystallinity observed for the perovskite in the *in situ* XRD experiments.^27−30^ Notably, the clear displacement of the PL distribution indicates that the expansion was isotropic, as broadening of the peak was minimal and no shoulders were observed.

**Figure 4:**
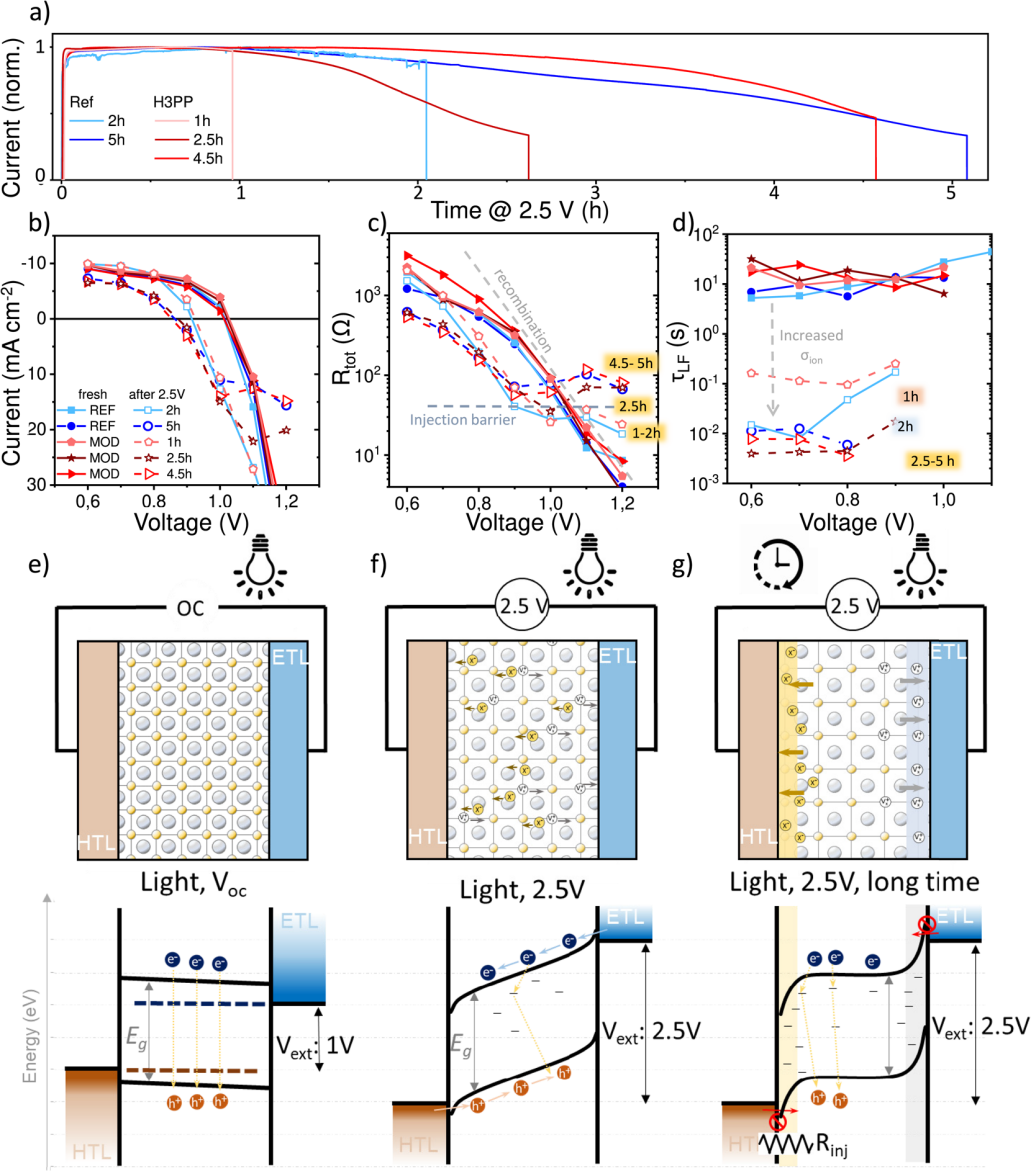
(a) Current evolution after applying 2.5 V bias for 2 REF and 3 MOD cells stopped at different times for EIS study. (b) J-V, (c) *R*_tot_ and (d) τ_LF_ extracted from EIS. Scheme of the ionic redistribution (top) and band alignment (bottom) in the PSC (e) at OC, (f) at 2.5 V, and (g) after long time at 2.5 V.

We also investigated the impact of an accelerated test with 2.5 V bias and 0.4 sun (Figure 4a) on the electrochemical properties. We performed EIS analyses on PSCs before (Figure 4e) and after stress with durations ranging from 1 to 5 h (Figure 4f–g). A direct correlation was found between the length of the 2.5 V stress and decreased recombination resistance (*R*_*rec*_) (Figure 4c) that reduced the *V*_*oc*_ and FF (Figure 4b). Furthermore, the accelerated stress resulted in the formation of a constant resistance at measured voltages beyond *V*_*oc*_, indicating a transition of *R*_*tot*_ from the *R*_*rec*_ signal to an additional voltage-independent process, identified below as a charge injection resistance (*R*_*inj*_). Interestingly, we observed an increase in ionic conductivity (σ_*ion*_ = ion density × mobility) up to 3 orders of magnitude after stress tests, consistent with an expanded lattice (Figure 4f–g). Subjecting the PSCs to 2.5 V bias resulted in a progressive reduction of the low frequency lifetime (τ_*LF*_, Figure 4d), that has been directly related to ionic conductivity.^17,18^ τ_*LF*_ is the product of the *R*_*LF*_ and the low frequency capacitance (τ_*LF*_ = *R*_*tot*_·*C*_*LF*_) obtained with the equivalent circuit in Figure S4b. Over time, the mobile ions can accumulate at the interface of the perovskite and create a large *R*_*inj*_ between 30 Ω and 118 Ω (Figure 4c–g). This barrier also provoked a saturated reverse current in the *J–V* curves, and we credit the accumulation of ions as the origin of the gradual current decrease during 2.5 V accelerated tests (Figures 3a and 4a). Additionally, we observed this ionic-injection barrier by *in situ* PL measurements (Figure S11). At the point where an ionic barrier is most likely being formed, we discerned a redshift of the PL center (lower bandgap) together with an increased PL intensity. We attribute this response to a reduced internal electric field in the perovskite film caused by increasing series *R*_*inj*_. The reduced internal field caused a partial relaxation of the bias-induced strain and more radiative recombination as fewer carriers were extracted from the perovskite layer. The combined effect of the interfacial barrier and the trap defect formation by ion migration resulted in enhanced recombination and thereby led to the observed charge loss and *V*_oc_ drop. Eventually, the large lattice expansion, in conjunction with the increased mobile ion formation and accumulation, resulted in short circuits of several devices, as observed repeatedly.

In summary, EIS analyses revealed that the progressive lattice expansion, observed under accelerated stability analyses, caused degradation and an increase of σ_ion_ evidenced by the change in τ_*LF*_ (Figure 4f). For longer accelerated tests, the lattice expanded further, thereby increasing the σ_ion_. These mobile ions accumulated at the interfaces of the perovskite, creating charge transfer and injection barriers at the selective contacts (Figure 4e). Both the expansion of the lattice and the eventual creation of an interfacial barrier could be corroborated by PL results. Both the MOD and REF devices investigated in these studies showed a similar behavior.

In conclusion, we repeatedly monitored the strain modulation observed in halide perovskites (HPs) during accelerated stability tests conducted on PSCs under continuous light irradiation and bias-voltage. We accomplished a small initial compression of the HP lattice via an additive engineering strategy incorporating the organic molecule H3pp within the HP. We then conducted accelerated stability tests to the reference (REF) and modified (MOD) PSCs while tracking the temporal evolution of the HP lattice and the device response. In our studies, we employed *in situ* X-ray diffraction, photoluminescence and quasi *in situ* electrochemical impedance spectroscopy analyses. Our results show a clear correlation with the strain of the HP crystal lattice, with accompanied band gap shift and solar cell device degradation, observed through the current decay. We observed lattice expansion in both REF and MOD HP during stability analyses independently of the initially compressed lattice parameters of the MOD samples. The outcome supports that ensuring lattice compression via additive engineering in the as-prepared HP samples effectively enhances the PSC stability. However, this does not guarantee long-term stability. To our knowledge, this is the first report showing the evolution of the lattice dynamics across a performance-monitored, accelerated stability analysis.

## Data Availability

The data underlying this study are openly available in CORA RDR (or CORA.Repositori de Dades de Recerca) at https://doi.org/10.34810/data1898. Please find the private sharing link to the dataset on https://dataverse.csuc.cat/privateurl.xhtml?token=2c83da64-9e66-4a6a-8820-fdf8fc59626e for preview of the unpublished dataset.

## Supporting Information Available

Experimental methods, Evaluation of thin Au layers, *In-situ* XRD sample setup, XRD fittings, statistical analysis and volume estimations, EIS and ionic conductivity before stress, Residual stress analysis by XRD Chi-scans, Bias-dependency of lattice changes, reversibility and corresponding time scales on current decays, XRD, and PL, Reproducibility of characteristic current decay, Declaration of irreversible degradation after long stress, PL over time under bias. (PDF)

## References

[ref1] Liu D.; Luo D.; Iqbal A. N.; Orr K. W. P.; Doherty T. A. S.; Lu Z.-H.; Stranks S. D.; Zhang W. Strain analysis and engineering in halide perovskite photovoltaics. Nat. Mater. 2021, 20 (10), 1337–1346. 10.1038/s41563-021-01097-x. 34531574

[ref2] Zhang H.; Park N.-G. Strain Control to Stabilize Perovskite Solar Cells. Angew. Chem., Int. Ed. 2022, 61 (48), e202212268 10.1002/anie.202212268. 36121756

[ref3] Liu Y.; Sumpter B. G.; Keum J. K.; Hu B.; Ahmadi M.; Ovchinnikova O. S. Strain in Metal Halide Perovskites: The Critical Role of A-Site Cation. ACS Applied Energy Materials 2021, 4 (3), 2068–2072. 10.1021/acsaem.1c00225.

[ref4] Meng W.; Zhang K.; Osvet A.; Zhang J.; Gruber W.; Forberich K.; Meyer B.; Heiss W.; Unruh T.; Li N.; et al. Revealing the strain-associated physical mechanisms impacting the performance and stability of perovskite solar cells. Joule 2022, 6 (2), 458–475. 10.1016/j.joule.2022.01.011.

[ref5] Wang L.; Song Q.; Pei F.; Chen Y.; Dou J.; Wang H.; Shi C.; Zhang X.; Fan R.; Zhou W.; et al. Strain Modulation for Light-Stable n–i–p Perovskite/Silicon Tandem Solar Cells. Adv. Mater. 2022, 34 (26), 2201315 10.1002/adma.202201315. 35435280

[ref6] Zhang L.; Luo G.; Zhang W.; Yao Y.; Ren P.; Geng X.; Zhang Y.; Wu X.; Xu L.; Lin P.; et al. Strain Regulation and Defect Passivation of FA-Based Perovskite Materials for Highly Efficient Solar Cells. Advanced Science 2024, 11 (7), 2305582 10.1002/advs.202305582. 38064168 PMC10870053

[ref7] Kim H.; Lee J. W.; Han G. R.; Kim S. K.; Oh J. H. Synergistic Effects of Cation and Anion in an Ionic Imidazolium Tetrafluoroborate Additive for Improving the Efficiency and Stability of Half-Mixed Pb-Sn Perovskite Solar Cells. Adv. Funct. Mater. 2021, 31 (11), 2008801 10.1002/adfm.202008801.

[ref8] Wang Q.; Jiang X.; Peng C.; Zhang J.; Jiang H.; Bu H.; Yang G.; Wang H.; Zhou Z.; Guo X. Regulating the lattice strain in perovskite films to obtain efficient and stable perovskite solar cells. Chemical Engineering Journal 2024, 481, 148464 10.1016/j.cej.2023.148464.

[ref9] Li Z.; Li X.; Feng X.; Chen X.; Chen J.; Cui X.; La S.; Yuan Z.; Zhang Z.; Wang X.; et al. Strain Control of Mixed-Halide Wide-Bandgap Perovskites for Highly Efficient and Stable Solar Cells. Solar RRL 2023, 7 (22), 2300615 10.1002/solr.202300615.

[ref10] Chen B.-A.; Lin J.-T.; Suen N.-T.; Tsao C.-W.; Chu T.-C.; Hsu Y.-Y.; Chan T.-S.; Chan Y.-T.; Yang J.-S.; Chiu C.-W.; et al. In Situ Identification of Photo- and Moisture-Dependent Phase Evolution of Perovskite Solar Cells. ACS Energy Letters 2017, 2 (2), 342–348. 10.1021/acsenergylett.6b00698.

[ref11] Fransishyn K. M.; Kundu S.; Kelly T. L. Elucidating the Failure Mechanisms of Perovskite Solar Cells in Humid Environments Using In Situ Grazing-Incidence Wide-Angle X-ray Scattering. ACS Energy Letters 2018, 3 (9), 2127–2133. 10.1021/acsenergylett.8b01300.

[ref12] Schelhas L. T.; Christians J. A.; Berry J. J.; Toney M. F.; Tassone C. J.; Luther J. M.; Stone K. H. Monitoring a Silent Phase Transition in CH3NH3PbI3 Solar Cells via Operando X-ray Diffraction. ACS Energy Letters 2016, 1 (5), 1007–1012. 10.1021/acsenergylett.6b00441.

[ref13] Di Girolamo D.; Phung N.; Kosasih F. U.; Di Giacomo F.; Matteocci F.; Smith J. A.; Flatken M. A.; Köbler H.; Turren Cruz S. H.; Mattoni A.; et al. Ion Migration-Induced Amorphization and Phase Segregation as a Degradation Mechanism in Planar Perovskite Solar Cells. Adv. Energy Mater. 2020, 10 (25), 2000310 10.1002/aenm.202000310.

[ref14] Kim D.; Yun J. S.; Sharma P.; Lee D. S.; Kim J.; Soufiani A. M.; Huang S.; Green M. A.; Ho-Baillie A. W. Y.; Seidel J. Light- and bias-induced structural variations in metal halide perovskites. Nat. Commun. 2019, 10 (1), 444 10.1038/s41467-019-08364-1. 30683878 PMC6347646

[ref15] Xie H.; Wang Z.; Chen Z.; Pereyra C.; Pols M.; Gałkowski K.; Anaya M.; Fu S.; Jia X.; Tang P.; et al. Decoupling the effects of defects on efficiency and stability through phosphonates in stable halide perovskite solar cells. Joule 2021, 5 (5), 1246–1266. 10.1016/j.joule.2021.04.003.

[ref16] Karimipour M.; Paingott Parambil A.; Tabah Tanko K.; Zhang T.; Gao F.; Lira-Cantu M. Functionalized MXene/Halide Perovskite Heterojunctions for Perovskite Solar Cells Stable Under Real Outdoor Conditions. Adv. Energy Mater. 2023, 13 (44), 2301959 10.1002/aenm.202301959.

[ref17] Neukom M. T.; Schiller A.; Züfle S.; Knapp E.; Ávila J.; Pérez-del-Rey D.; Dreessen C.; Zanoni K. P. S.; Sessolo M.; Bolink H. J.; et al. Consistent Device Simulation Model Describing Perovskite Solar Cells in Steady-State, Transient, and Frequency Domain. ACS Appl. Mater. Interfaces 2019, 11 (26), 23320–23328. 10.1021/acsami.9b04991. 31180209

[ref18] Lammar S.; Escalante R.; Riquelme A. J.; Jenatsch S.; Ruhstaller B.; Oskam G.; Aernouts T.; Anta J. A. Impact of non-stoichiometry on ion migration and photovoltaic performance of formamidinium-based perovskite solar cells. Journal of Materials Chemistry A 2022, 10 (36), 18782–18791. 10.1039/D2TA04840J.

[ref19] Tsai H.; Asadpour R.; Blancon J.-C.; Stoumpos C. C.; Durand O.; Strzalka J. W.; Chen B.; Verduzco R.; Ajayan P. M.; Tretiak S.; et al. Light-induced lattice expansion leads to high-efficiency perovskite solar cells. Science 2018, 360 (6384), 67–70. 10.1126/science.aap8671. 29622649

[ref20] Baumann F.; Raga S. R.; Lira-Cantú M. Monitoring the stability and degradation mechanisms of perovskite solar cells by in situ and operando characterization. APL Energy 2023, 1 (1), 011501 10.1063/5.0145199.

[ref21] Tan W. L.; McNeill C. R. X-ray diffraction of photovoltaic perovskites: Principles and applications. Applied Physics Reviews 2022, 9 (2), 021310 10.1063/5.0076665.

[ref22] Kundu S.; Kelly T. L. In situ studies of the degradation mechanisms of perovskite solar cells. EcoMat 2020, 2 (2), e12025 10.1002/eom2.12025.

[ref23] Jeon N. J.; Noh J. H.; Yang W. S.; Kim Y. C.; Ryu S.; Seo J.; Seok S. I. Compositional engineering of perovskite materials for high-performance solar cells. Nature 2015, 517 (7535), 476–480. 10.1038/nature14133. 25561177

[ref24] Eperon G. E.; Stone K. H.; Mundt L. E.; Schloemer T. H.; Habisreutinger S. N.; Dunfield S. P.; Schelhas L. T.; Berry J. J.; Moore D. T. The Role of Dimethylammonium in Bandgap Modulation for Stable Halide Perovskites. ACS Energy Letters 2020, 5 (6), 1856–1864. 10.1021/acsenergylett.0c00872.

[ref25] Jiang J.; Xiong M.; Fan K.; Bao C.; Xin D.; Pan Z.; Fei L.; Huang H.; Zhou L.; Yao K.; et al. Synergistic strain engineering of perovskite single crystals for highly stable and sensitive X-ray detectors with low-bias imaging and monitoring. Nat. Photonics 2022, 16 (8), 575–581. 10.1038/s41566-022-01024-9.

[ref26] Xue D.-J.; Hou Y.; Liu S.-C.; Wei M.; Chen B.; Huang Z.; Li Z.; Sun B.; Proppe A. H.; Dong Y.; et al. Regulating strain in perovskite thin films through charge-transport layers. Nat. Commun. 2020, 11 (1), 1514 10.1038/s41467-020-15338-1. 32251277 PMC7090003

[ref27] Boyer-Richard S.; Katan C.; Traoré B.; Scholz R.; Jancu J.-M.; Even J. Symmetry-Based Tight Binding Modeling of Halide Perovskite Semiconductors. J. Phys. Chem. Lett. 2016, 7 (19), 3833–3840. 10.1021/acs.jpclett.6b01749. 27623678

[ref28] Shohonov D. A.; Migas D. B.; Filonov A. B.; Borisenko V. E.; Takabe R.; Suemasu T. Effects of lattice parameter manipulations on electronic and optical properties of BaSi2. Thin Solid Films 2019, 686, 137436 10.1016/j.tsf.2019.137436.

[ref29] Prasanna R.; Gold-Parker A.; Leijtens T.; Conings B.; Babayigit A.; Boyen H.-G.; Toney M. F.; McGehee M. D. Band Gap Tuning via Lattice Contraction and Octahedral Tilting in Perovskite Materials for Photovoltaics. J. Am. Chem. Soc. 2017, 139 (32), 11117–11124. 10.1021/jacs.7b04981. 28704048

[ref30] Zhu C.; Niu X.; Fu Y.; Li N.; Hu C.; Chen Y.; He X.; Na G.; Liu P.; Zai H.; et al. Strain engineering in perovskite solar cells and its impacts on carrier dynamics. Nat. Commun. 2019, 10 (1), 815 10.1038/s41467-019-08507-4. 30778061 PMC6379394

