# Supplementary material for: Strain in Halide Perovskites and Solar Cell Stability: Accelerated Stress Tests under Bias Voltage
Source: ACS Energy Lett. 2024 Dec 26;10(1):476–83. doi: 10.1021/acsenergylett.4c02822 (PMC11922313; doi:10.1021/acsenergylett.4c02822)
Supplement: Supplementary file 1 — nz4c02822_si_001.pdf [file nz4c02822_si_001.pdf]

Supplementary Information

STRAIN IN HALIDE PEROVSKITES AND SOLAR CELL STABILITY: ACCELERATED STRESS TESTS UNDER BIAS VOLTAGE

Fanny Baumann[1], Masoud Karimipour[1], Jessica Padilla-Pantoja[1], Emigdio Chávez Angel[1], Jose Manuel Caicedo Roque[1], Rémy Pouteaux[1], Alex Alcalá Ibarra[1], Sonia R. Raga[1*], José Santiso[1*] and Monica Lira-Cantu[1*]

1. *Catalan Institute of Nanoscience and Nanotechnology (ICN2), CSIC and the Barcelona Institute of Science and Technology (BIST), Building ICN2, Campus UAB, E-08193 Bellaterra, Barcelona, Spain*

Corresponding author emails: monica.lira@icn2.cat, sonia.ruiz@icn2.cat, jose.santiso@icn2.cat

Experimental Methods

Device Fabrication:

Materials for perovskite and hole transport layer deposition. N, N-dimethylformamide (DMF, anhydrous, 99.8%), dimethyl sulfoxide (DMSO, anhydrous, 99.8%), chlorobenzene (anhydrous, 99.8%), acetonitrile (anhydrous, 99.8%), isopropanol (99.97%, anhydrous), and 4-tert-butylpyridine (TBP, 96%) were received from Merck Co. lead(II) bromide ($PbBr_2$, 99.99%), lead (II) iodide (PbI_2, 99.99%), formamidinium iodide (FAI), rubidium iodide (RbI, 99.99%), methylammonium iodide (MAI), and cesium iodide (CsI, 99.99%) were purchased from TCI chemicals. Spiro-OMeTAD (Spiro) was purchased from Xi'an Polymer Light Technology Corp. All chemicals are used as received without any further purification. 3-phosphonopropionic acid (H3pp), tetrapropylammonium hydroxide (TPAOH, 58%), tetrabutylammonium hydroxide (TBAOH, 45%), tetramethylammonium hydroxide (TMAOH, 25%) were purchased from Sigma-Aldrich.

Perovskite Solution. 950, 190, 60 and 30 µL of $FAPbI_3$, $MAPbBr_2I$, CsI and RbI solutions were mixed in a vial to form the $Rb_{0.05}Cs_{0.05}(FA_{0.83}MA_{0.17})_{0.95}Pb(I_{0.83}Br_{0.17})_3$ perovskite solution. 1.5 M PbI_2 and $PbBr_2$ in DMF:DMSO (4:1 vol. ratio) were prepared separately and the solutions were heated to 150°C for 5 min. 0.2 g of FAI and 0.05 g of MAI were weighed in separate vials and then 1745 mg PbI_2 and 365 mg of $PbBr_2$ prepared solutions were added to them, respectively, and shaken until they were dissolved completely to form $FAPbI_3$, and $MAPbBr_2I$ solutions, respectively. Stock solutions of dopants were made from 0.1 g of RbI in 375 µL DMSO and CsI in 287 µL DMSO.

H3pp modification. For "MOD" or "modified" devices with H3pp:HP, 4.5 µl of H3pp stock solution (20 mg/0.5ml DMF) was injected into 0.5 ml of perovskite solution and the vial was shaken for 20 s.

HTL Solution. 2,2',7,7'-Tetrakis[N,N-di(4-methoxyphenyl)amino]-9,9'-spirobifluorene (Spiro-OMeTAD, 60 mg, Aldrich) was dissolved in chlorobenzene (0.596 mL, CB). 4-tert-Butylpyridine (23µl, TBP) and tris(2-(1Hpyrazol-1-yl)-4-tert-butylpyridine) cobalt(III) tri[bis- (trifluoromethane)sulfonimide] (FK209, Aldrich) precursor solution (4.5 µl from 375 mg in 1 mL acetonitrile) and Lithium bis(13.5 µl, trifluoromethanesulfonyl) imide (Li-TFSI, Aldrich) precursor solution (100 mg in 378 mL acetonitrile) was added to the solution just before the deposition.

Device fabrication. Devices were prepared in n-i-p stacking with structure FTO (0.5 µm)/ TiO_2-c (80 nm)/ TiO_2-mp (150 nm)/ perovskite (400-450 nm)/ spiro (200 nm)/ Au (70 nm or 70 nm +7 nm). FTO (16 Ω/cm^2) substrates (2.5 cm×1.5 cm) were etched using Zinc powder and HCl 4M, brushed with

10% Hellmanex^{TM} and then placed in a sonicating bath with sequential cleaning using 2% Hellmanex^{TM} solution, Acetone, Isopropanol, for 30 min, 10 min, 15 min, respectively, followed by UV/Ozone cleaner for 20 min. Solution of 9 mL ethanol (96 %), 0.4 mL of acetyl acetone, and 0.6 mL of Titanuim di-isopropoxide bis-acetyl acetonate, was deposited through O_2 spray-pyrolysis on 450°C pre-heated substrates to obtain a 20 nm thick TiO_2 blocking compact layer. The mesoporous TiO_2 layer (TiO_2-mp) was deposited from solution of 1mg TiO_2 paste (nanoparticles 30 nm) diluted in 6 g of ethanol, deposited by spin-coated at 5000 rpm for 20 s, dried at 85°C and thereafter annealed at 450°C for 30 min and cooled down naturally. Before being moved to a controlled N_2 atmosphere, samples were exposed to UV-Ozone for 25 min and transferred immediately to the glove box, maintained at 18-20°C (H_2O: 1-1.5 ppm, O_2:3-6 ppm), for perovskite, interface modification and HTL layer deposition. 0.050 mL perovskite solution was spread over the substrates and span at 2000 rpm for 10 s and 6000 rpm for 30 s, and after 15 s of the second step, 0.2 mL of CB was injected instantly to the center of the substrate and after spinning, the film was quickly annealed at 100°C for 20 min. The HTL layer was deposited by drop casting 35 µL of spiro solution spinning at 3800 rpm for 20 s. The 70 nm gold contact was deposited using a high vacuum PVD system with pressure below 8×10^{-7} atm.

For *in-situ* XRD measurement the 80 nm finger electrode was deposited by PVD followed by a second step of EBPVD of the thin 7 nm gold as described also in the main article.

Characterization:

Current Density – Voltage (JV) measurements. JV scans presented were performed under a halogen light source in a class AAA AM1.5G Helios Solar Simulator calibrated to 1 sun on a device active area of 0.16 cm^2. Scans forward from -0.1 to 1.2 V and reverse from 1.2 to -0.1 V were performed at scan speed 100 mV/s with 20 mV steps using a Keithley SourceMeter.

X-ray diffraction (XRD) measurements. XRD measurements were performed in a four-circle geometry Diffractometer *Malvern-Panalytical X'pert Pro MRD*, in a parallel beam optics configuration (Parabolic Mirror + D.S. Slit ¼° + 2mm mask).[1] This diffractometer has a horizontal ω-2θ goniometer (320 mm radius) and uses a ceramic X-ray tube with Cu Kα anode (λ = 1.5418 Å) as source. A Parabolic Mirror was applied in the incident beam and the equipment employs a fast X-ray *PIXcel* area detector, which is a detector based on *Medipix2* technology with a 256 x 256 pixels array.

X-ray diffraction (XRD) Chi-scan measurements. To achieve parallel beam optics for the Chi-scan we utilized several optical modifications to the same equipment mentioned above, including point-focus Cu Kα source with a polycapillary lens in the primary beam path, and a parallel plate collimator, D.S. Slit ¼° slits and Ni filter in the diffracted optics before the detector.

Photoluminescence (PL) spectroscopy measurements. Samples were measured with a 453.6 nm pulsed laser (PDL 820, ~40 MHz) of area ~1 mm^2 from the back(glass) side of the cell at the magic angle (54.7°). The measurements were carried out using a fluorescence spectrometer PicoQuant FluoTime 300 as sold (detector TimeHarp 260, s-t-n ratio > 32000:1) with adaptation to flow nitrogen in the measurement chamber and connect the solar cells electronically with a Keithley SourceMeter and in-house software. The electrical circuit was used to perform JV scans, apply accelerative voltage bias stress and monitor the current output, while simultaneously acquiring PL spectra. Each steady state PL emission spectrum was acquired using an identical EasyTau script where acquisition took ~30 s, followed by 30 s dark rest, for a total cycle time of 1 minute and 2 s of laser on/off conditions.

Electrochemical Impedance Spectroscopy (EIS). PAIOS from FLUXiM AG was used for EIS and some additional JV and accelerated tests. During EIS devices were kept at constant illumination of ~0.5 sun white LEDs spectra (approximated from current output comparison with Helios Solar Simulator), in

order to simulate the conditions in the *in-situ* XRD holder, and at nitrogen flow. EIS of devices were measured at V_{AC} perturbation 10 mV, at V_{DC} voltages between 0.6 V to 1.2 V and at frequencies between 4 MHz to 0.03 Hz.

Data processing. Processing of presented data was performed from raw data in excel sheets or raw data (scripts and raw data are openly available in CORA Repositori de Dades de Recerca at https://doi.org/10.34810/data1898.[2]) Analysis was done with Python, including open source libraries Matplotlib, Pandas, Numpy, Math, and Scipy. Spectral Analysis was performed by Matlab™ (licensed). Other plots were made using Orange Data Mining™,[3] and/or OriginLab™ (licensed). Equivalent circuit fittings of EIS were performed using ZView™ (licensed). Syre.ai (https://syre.ai/, licensed) was used to perform additional analysis of large sets of data.

S1. Thin Au evaluation.

Thin metal layers tend to aggregate over time (Figure S1), leading to increased resistivity. Thus, to determine the quality of the thin Au film, scanning electron microscopy (SEM) images were taken on selected films. Depending on the quality of deposition, in our case electronic device grade Electron-Beam Physical Vapor Deposition (EBPVD), we found that the thin films lost their ability to conduct the electric field over time due to island formation. Figure S1a-d shows pictures of high-quality film taken after deposition and before *in-situ* XRD experiments. Figure S1e shows a thin Au film after 2.5 V bias test *in-situ* XRD, demonstrating how the gold aggregated partially but remained connected. Depending on the evaporation quality of the Au layer, we estimated that the layer can maintain/keep a connection layer for at least 6-7 days, as seen in S1f and S1e (where EBPVD was less successful). We concluded that most connected layers can last up to 20 days (S1g), after that, the Au islands become isolated, as shown in S1h. 2-point-probe measurements on a thin film revealed a resistance ~0.5 kΩ at a distance of ~ 1 mm.

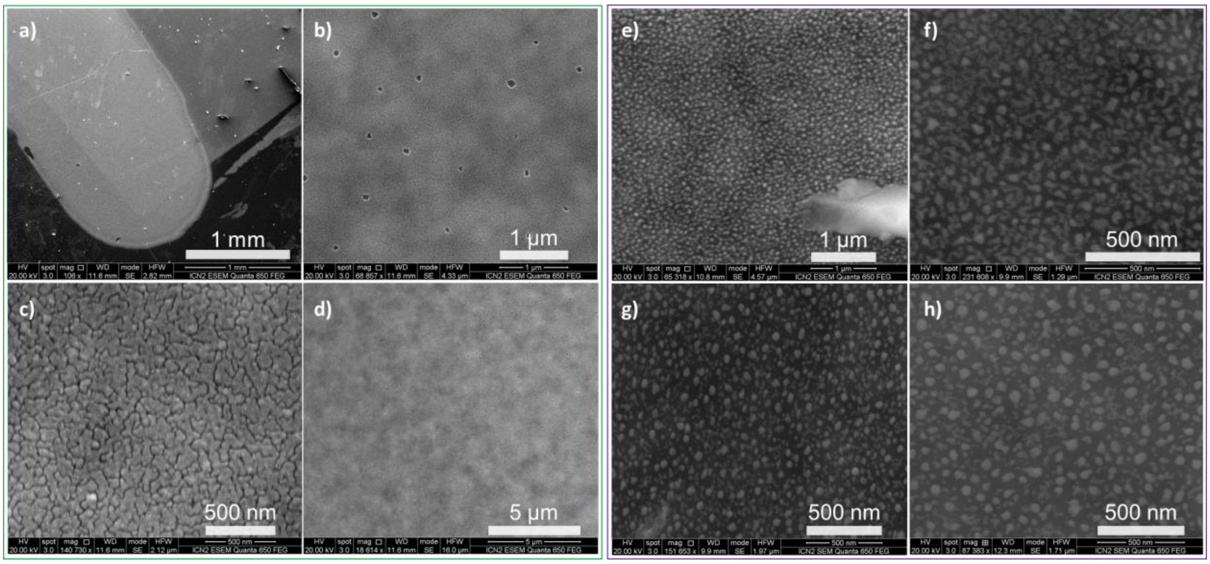

Figure S1. SEM images. a-d) Freshly evaporated thin Au on with modified top-electrode. a) SEM image of gold finger, thin gold and uncovered spiro-MeOTAD surface, b-d) thin Au at different sites and scales, specifically: c) Successful deposition of 7-10 nm thin Au on PSC controlled before XRD experiments 1 day after deposition. e-h) Thin Au layer with different degrees of island formation. e) PSC after in-situ XRD bias, 10 days after evaporation, 8 days after experiment (acceptable for conduction). f) poorly

evaporated film 6 days after evaporation (not tested or presented in results), g) decent evaporation over 20 days after deposition. h) Same as g), 50 days after EBPVD.

S2. *In-situ* XRD sample set-up.

Figure S2 shows images of the sample mount used for *in-situ* XRD analysis. The sample chamber (inside the dome, volume approximate 57 cm^3) was kept under room temperature N_2 flow of under 100 ppm O_2 (long operating value <7 ppm), and undetectable H_2O.

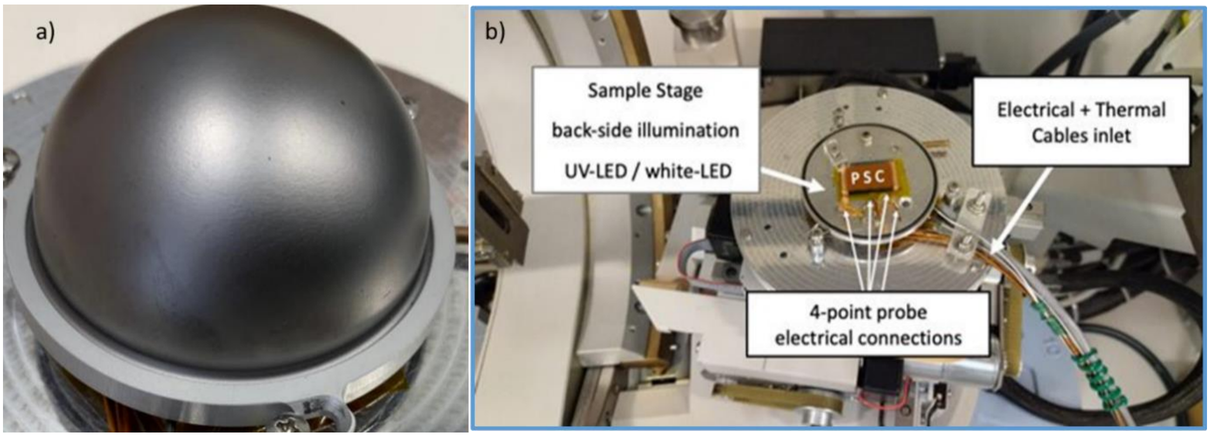

Figure S2. Images of a) graphite dome (roughly 5.6 cm diameter) transparent to X-rays, and b) sample mount with labelled components.

S3. XRD fittings and volume estimations.

XRD scans were first background-corrected using an adaptation of ALS (Asymmetric Least Squares, GNU licence, see attached code for references)[2]. A standard Pseudo-Voigt function was fitted to identified peak intervals, and if several intervals were identified for each peak the peak with the lowest standard deviation of the Gaussian component of the Pesudo-Voigt fitting was selected. The center 2θ of the peak was used, applying Bragg's law ($n\lambda = 2dsin(\theta)$) to calculate the out-of-plane interplanar spacing (d) and the lattice parameter *a* for the corresponding *hkl* (Miller index) reflection,[4] according to $d = \frac{a}{\sqrt{h^2 + k^2 + l^2}}$; where a cubic structure of cell parameter *a* is assumed.

To account for possible sample misalignments that induce subtle shifts in the peaks position in a XRD pattern and extract the most accurate cell parameter values the Nelson-Riley analysis was used for a larger collection of *hkl* reflections in a wider range of 2θ. This method consists of representing a linear regression of the equivalent cubic cell parameter values from a wide collection of *hkl* reflections against the $x = \frac{cos^2(\theta)}{\sin\theta} + \frac{cos^2(\theta)}{\theta}$ coordinate. The most accurate cell parameter is taken from the extrapolation at x = 0. We believe it reasonable to assume cubic structure as in the Nelson-Riley analysis none of the *hkl* reflections significantly deviated from the linear relationship (Figure 3d in main text), indicating that cubic structure was maintained during the accelerated test. Additionally, preferential orientation analysis showed non-distinguishable changes in the (002) and (001) XRD relative peak intensity (Figure S3.4d), demonstrating similar HP microstructure before, during, and after experiments.

In the scans with a shorter 2θ range for the in-situ XRD monitoring the Nelson-Riley approach was not possible. Instead, to account for any misalignment both initially between samples or during

experiments a correction of the obtained *a*-values were made using FTO as an internal reference by assuming that the substrate peak (FTO (110)) of the scan is identical for all samples and instances, as no changes are expected from the substrate. We adopted a relative correction with respect to FTO (110) peak, given the small range of 2θ included in the analysis. A more accurate correction would take into account the angular positions of the different *hkl* included in the analysis, but some preliminary tests gave differences in the order of 0.0001 Å, far below the accuracy of the measurement. Therefore, we decided for the linear correction for simplicity. As the reference value we used the average *a*-value of the FTO (110) obtained from the initial XRD scans of all samples included in the analysis. In time resolved plots all *a*-values were corrected according to the relative displacement of the scan, comparing the FTO (110) peak position (*a*-value) to the average FTO *a*-value obtained from the initial scans. This excluded observable misalignment effects.[1] The correction process and initial fitting values, available in Supporting Data (including analysis scripts),[2] are visualized in Figure S3.1, S3.2 and S3.3.

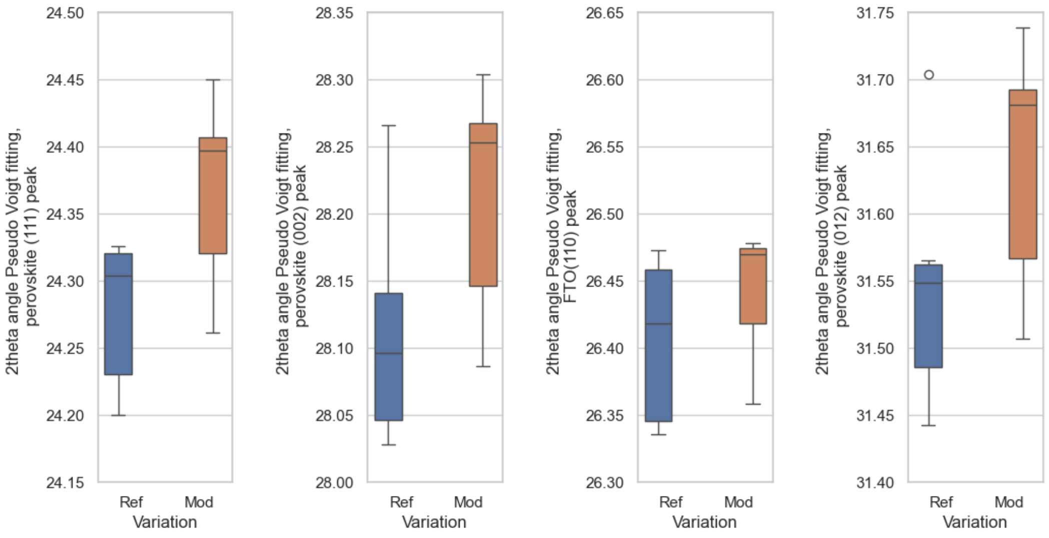

Figure S3.1. Initial 2θ positions as fitted by Pseudo-Voigt function. (111), (002), and (012), respectively observed at 2θ ≈ 24.30°, 28.10°, 31.53° for REF and 2θ ≈ 24.38°, 28.25°, 31.68° for MOD.

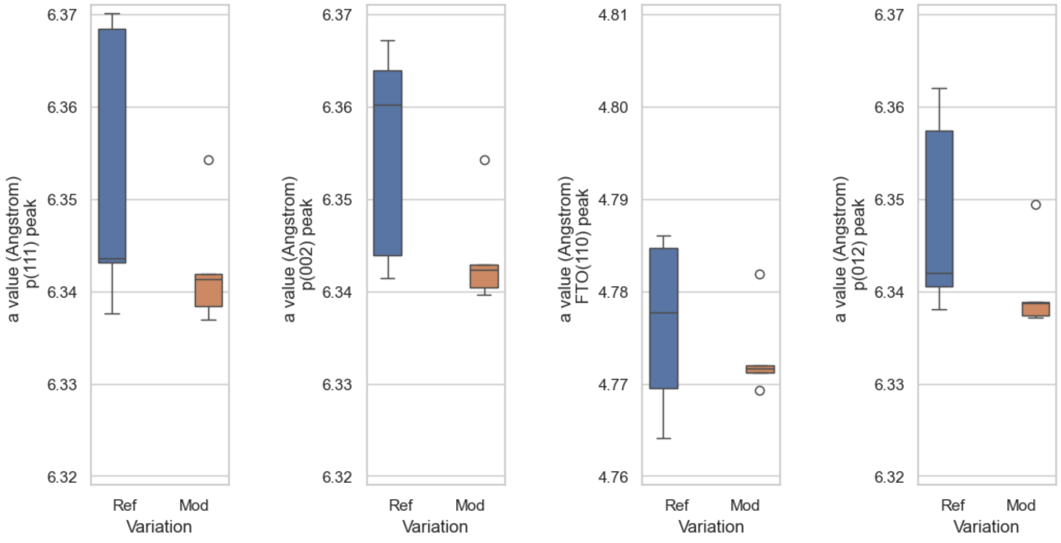

Figure S3.2. Raw a-values before alignment via relative a-value of FTO (110).

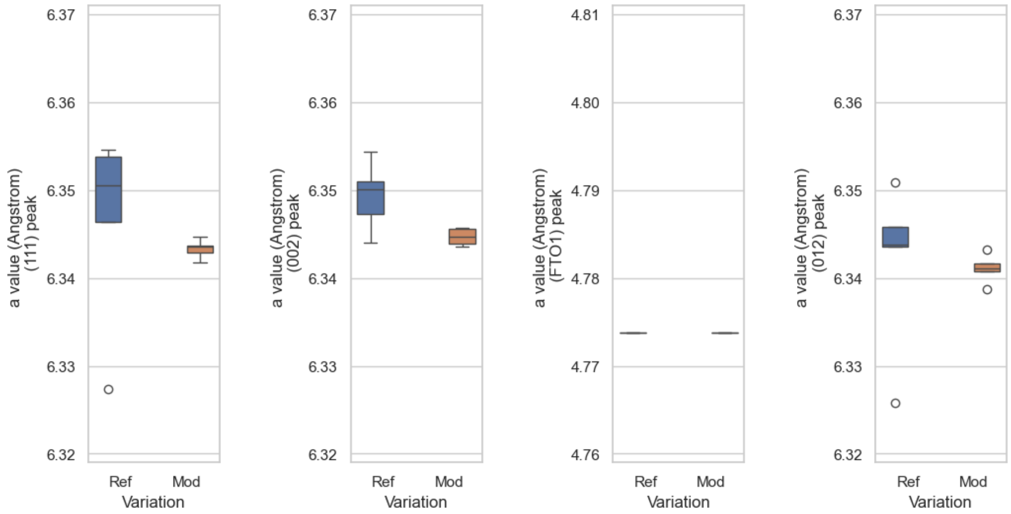

Figure S3.3. Final a-value of initial scans after aligning to FTO (110) via relative a-value change.

After observations of coherent shift in PL studies (See Figure 3b and S11), the linear relationship of the Nelson-Riley plot (Figure 3d) and the dismissal of residual strain effects (Figure S5), isotropic expansion was assumed. The initial unit cell volumes of the HP in the MOD and REF samples were calculated from the values obtained by Nelson-Riley regression (Figure 3d) as a^3 to ~252.3 Å³ (*a* = 6.319 Å) and ~253.3 Å³ (*a* = 6.327 Å), respectively. Similarly, the final volumes were calculated to ~253.4 Å³ (*a* = 6.328 Å) and ~254.5 Å³ (*a* = 6.337 Å).

a-values seen in the temporal plots (Figure 3c and S3.4) are estimates for comparison and should not be taken as the real values of the unit cell. The time series fitting including initial scans of in-situ XRD experiments and progression over time during the accelerated conditions are visualized in Figure S3.4.

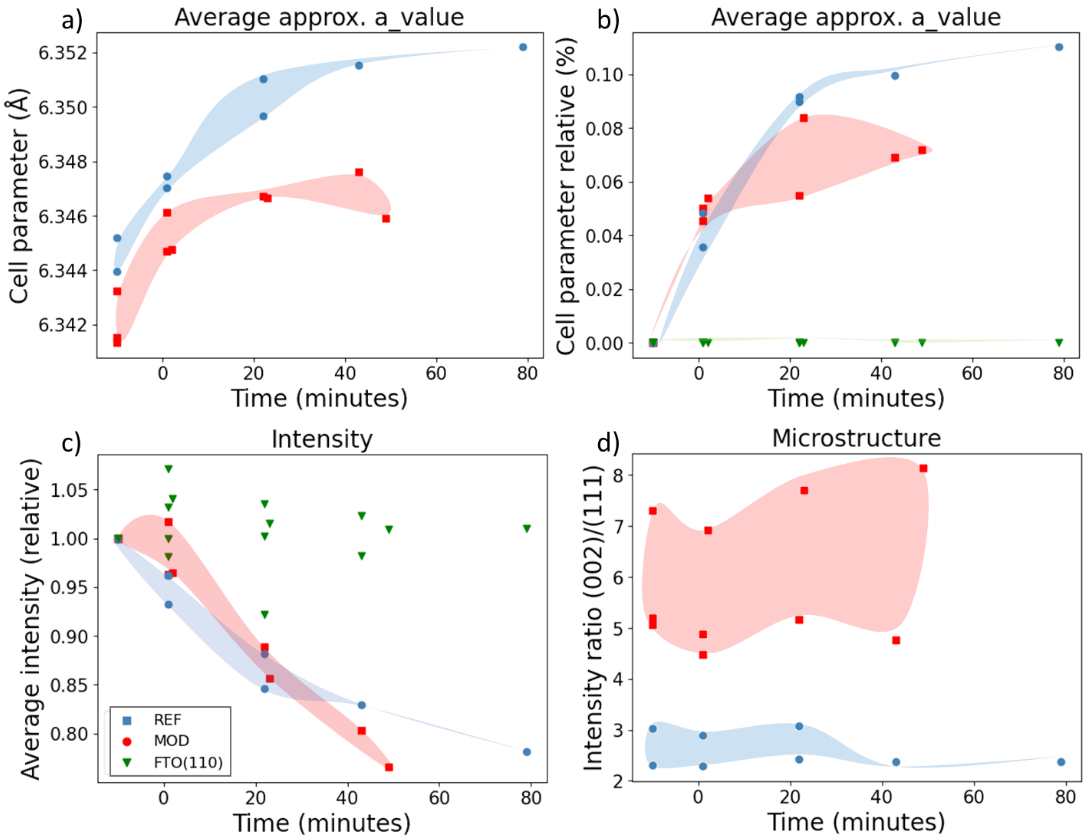

Figure S3.4. Perovskite lattice analysis over time with 2.5 V bias on MOD(red) and REF(blue) PSC. a)

average a-value from (111), (002) and (012) Bragg reflections b) relative progression of average a-value compared to initial values, including a-values extracted from FTO (110). c) Peak relative intensity over time (max of Pseudo-Voigt fitting. d) intensity of (002) divided by intensity of (111) to show the progression of relative peak height, showing the difference in grain microstructure (preferential orientation) and its preservation during the experiment.

S4. EIS and ionic conductivity before stress.

Characteristic times of the low frequency region in Figure S4a were calculated as $\tau_{LF} = R_{LF} \cdot C_{LF}$ using values obtained by fitting EIS signals to the equivalent circuit in Figure S4b. From the S4a we can notice that there was no significant difference in time constants at operating voltages before the stress-tests. At low voltages, a small difference can be noticed.

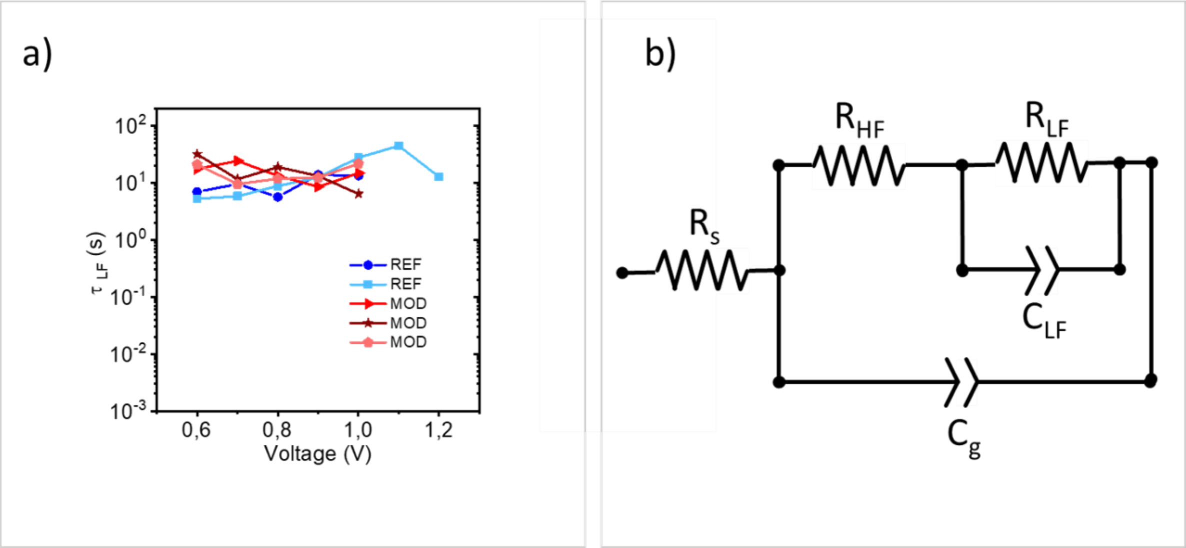

Figure S4. a) Low frequency characteristic times τ_{LF} coincided for initial EIS measurement of PSC with (MOD) and without (REF) the organic additive H3pp. b) Equivalent circuit used for EIS fittings to extract the low frequency resistance (R_{LF}) and the low frequency capacitance (C_{LF}).

S5. Residual strain analysis by XRD 2θ/ω Chi-scans.

To eliminate doubts about the residual strain in the HP film before and during experiments, we performed XRD 2θ/ω scans with χ variation (tilting of the surface in relation to the beam, Chi-scan) on one device with H3pp (MOD) and one REF from the experimental batches (Batch 8). This Chi-scan, with similar basis as an ω tilt (rocking curve) measurement, is a frequently used method in metallurgy and allows for seeing how the lattice plane distances vary when changing from out-of-plane to more in-plane angles.[1, 5] Parallel beam optics permits modifying the χ angle (the sideway tilt) of the sample without deforming the beam, with the drawback of widened peaks, making it more difficult to pinpoint the exact Bragg-reflection and rather obtaining an estimate of the tendency with changing χ. We acquired XRD scans of two HP peaks and two FTO peaks while tilting the surface of the samples in χ from 0 to 80 degrees. The corresponding interplanar distances are depicted in Figure S5 against the $\sin^2\chi$. The HP (220) reflection and HP (012) reflection did not show any positional variation in the whole range of χ analyzed (Figure S5). This confirmed that there was no residual strain visible from HP peak variations with χ angle, seen by the flat profile of the linear regression plot. However, the

FTO peaks of (110) and (200) showed a clear reduction of the interplanar distance with an almost linear dependence with $\sin^2\chi$. This is consistent with an out-of-plane expansion, and in-plane compression, typically coming from a larger thermal expansion of the glass substrate (60 to 85×10-7/°C), compared to that of FTO(FTO (35×10-7/°C). [5-7] Clearly, there is residual strain in the FTO layer which is directly adhered to the glass surface. However, this strain is not transmitted to the HP layer, as expected since the PSC is an intricate structure with several layers between the solid glass and the HP, including a mesoporous layer. This indicates that the effects observed in our experiments are not related to residual strain.

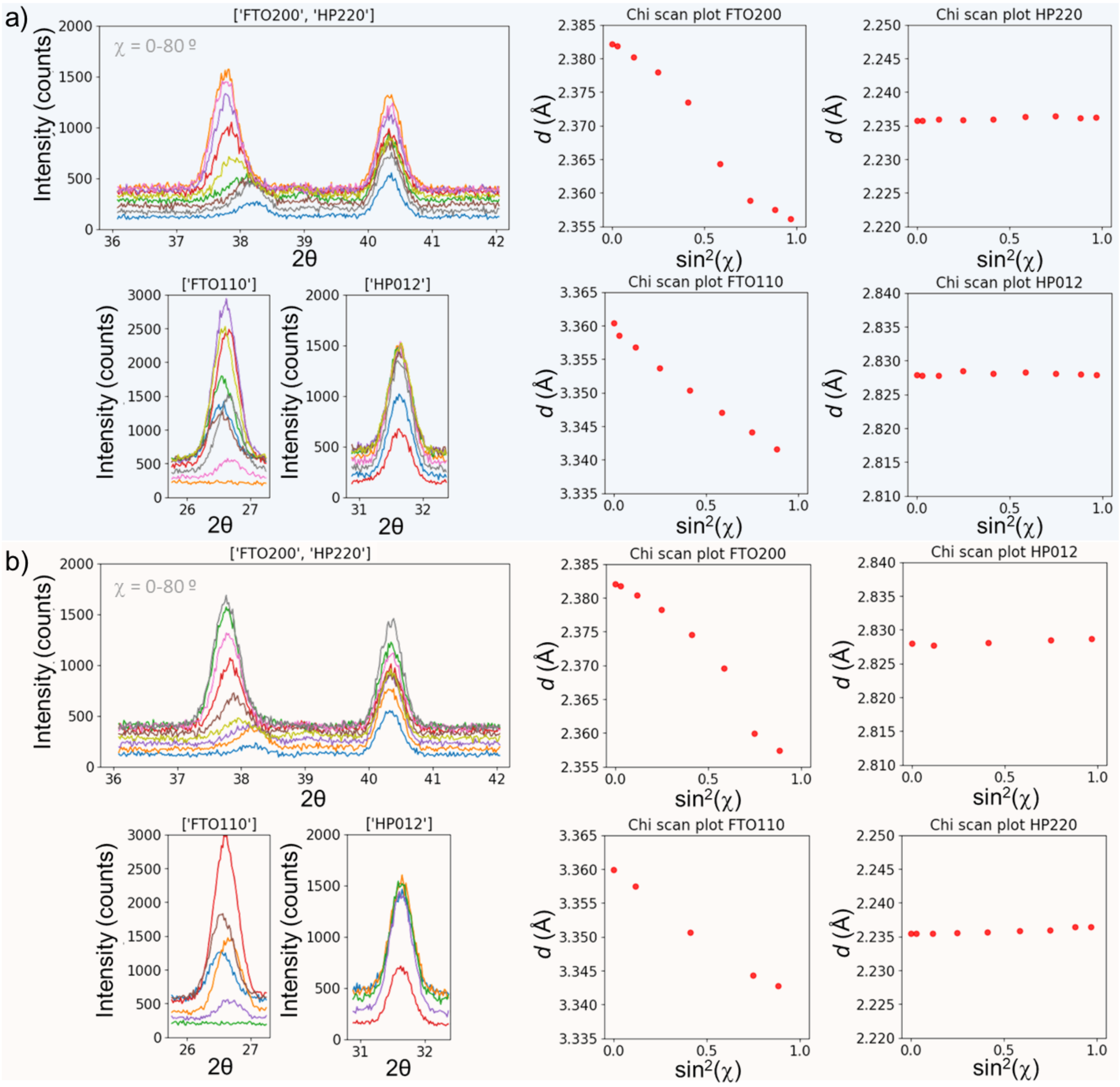

Figure S5. Investigation of residual stain in the HP and FTO film using XRD Chi-scan, on the left resulting $2\theta/\omega$ scans, on the right the interplanar distance d against $\sin^2(\chi)$. a) REF, b) MOD. Fittings were done by Pseudo Voigt function as described in S3.

S6. Reproducibility of characteristic current decay.

To ensure reproducibility and validate the relevance of the accelerated test, a number of similarly fabricated devices with finger and thin layer electrode (Figure S6a) and with standard 0.16 cm^2

electrode (Figure S6b) from different batches were tested under the same conditions. The measurements show a characteristic current decay.

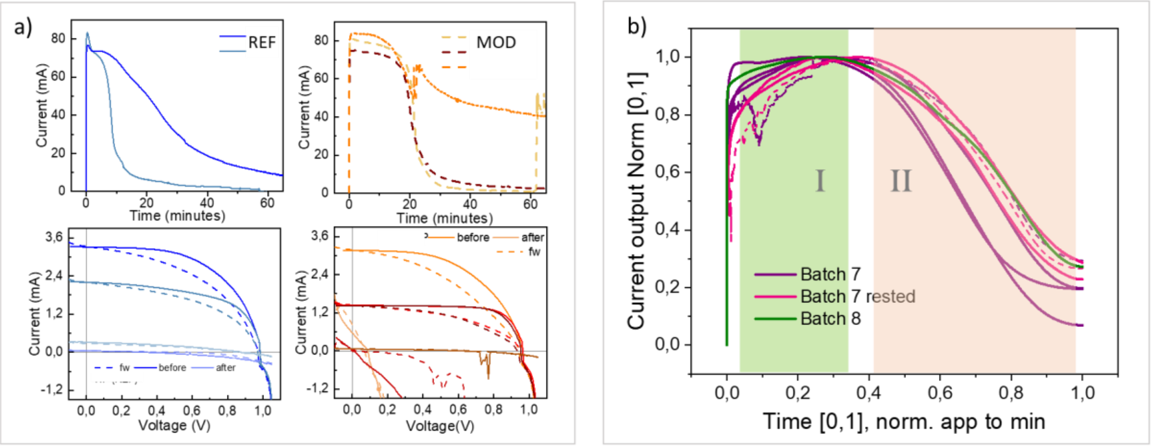

Figure S6. Reproducibility of characteristic current progression. All samples were measured in the XRD holder under N_2 flow at 2.5 V and 0.3 sun illumination. a) Customized Au electrode for in-situ XRD, left: REF right: MOD b) Standard PSC electrode, all REF, placed in XRD holder without measuring XRD, various batches.

Notice that for MOD, although reproducing the current progression, the risk for shorting at 2.5 V was high. No data points beyond shorting have been included in this work.

S7. Proof of bias-induced expansion and reversibility.

We ruled out the contribution of X-ray beam damage or illumination-induced heating by measuring a sample for 1 h at open circuit, subsequently applying high bias (Figure S7a). Additionally, we performed experiments ramping the voltage (Figure S7b).

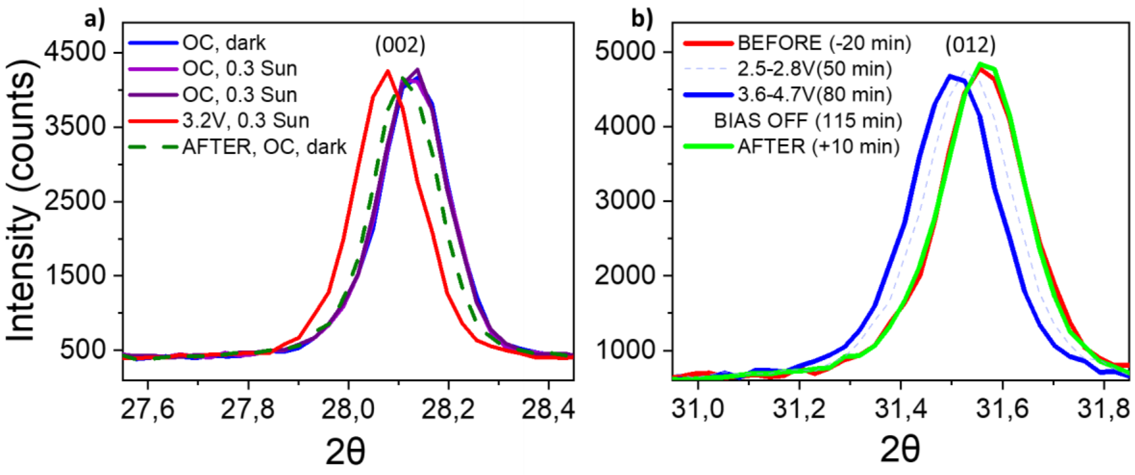

Figure S7. PSC in in-situ XRD. a) without bias (OC) at illumination and X-ray diffraction measurement for a total of 2 hours, followed by biasing to 3.2 V over 30 minutes, and recovery after 5 minutes. b) A gradual increase in voltage from MPP (0.8 V) while keeping the current at the initial increasing tendency. Recovery after over 100 minutes of bias reaching biases above 4.5 V.

When the PSC was kept without bias during X-ray exposure (measurements) and illumination of 0.3 sun, no shift was seen. Once a 3.2 V bias was applied, a shift was seen on the first subsequent scan. Later, when the bias voltage was removed and the cell was kept in the dark, the shift recovered almost completely. This indicates that the shift (expansion) occurs before loss in crystallinity. This observation is presented as additional proof that the shift was induced by the voltage bias. Additionally, monitoring the current during its first increase at high biases up to 4.8 V showed the same phenomenon, again an entirely reversible shift. Observed was only a slight reduction in the performance evaluating IV curve, a reduction largely recovered after 15-20 minutes in the dark under N_2 flow.

S8. Expansion at lower biases (1.2V) and corresponding timescales on current decay, XRD and PL.

During operation, a trade-off exists between the internal electric field and the photocarrier density inside the perovskite layer, dependent on the bias applied to the solar cell. Under short circuit (SC) conditions, one observes the highest current density and internal electric field, with the lowest concentration of accumulated charges. Under open circuit (OC) conditions, there is no current flow and thus, one observes the maximal accumulation of photo-induced charge carriers, and the internal field is minimal. The maximum power point tracking (MMP) mode, which is recommended by the updated ISOS protocols for the characterization of perovskite solar cells,[8] can be considered an intermediate condition between SC and OC, where the maximum proportion of charges are extracted at the maximum potential, giving us the maximum power as per $J * V$ (carrier density /area * potential of carriers). If the device is forward biased beyond the OC the additional injected carriers from the contacts add to the photogenerated ones, thereby increasing the total carrier density in the perovskite.

This equilibrium between carriers moving through the electric field and carriers trapped inside the device, has a strong effect on the stability of PSC. It is known that PSCs degrade faster under SC conditions and fully recover after the light is switched off, while under OC the PSCs do not recover but instead further decreases after the light is switched off ("drop-in-dark" effect).[9]

Alongside experiments applying 2.5 V voltage bias as presented in the main article, we have consequently performed experiments at 1.2 V to confirm that the phenomena observed is relevant at biases currently applied to solar cells under general characterization. In performance evaluating JV curves, the open circuit (OC) voltage of the cell is often between 1.0 and 1.1 V, therefore it is tempting to scan all the way to 1.2 V, something that has become common practise. Reproducing the XRD shift and current evolution under 1.2 V indicates that this phenomenon is occurring just above OC. If this phenomenon can occur below the OC voltage remains an open question.

Figure S8a shows the tendency of measurements at 1.2 V, a similar tendency as at 2.5 V but where PCE took over 6 times longer to decay. This was another motivation for performing XRD at accelerating 2.5 V. Normalizing the time from first maxima to first minima as seen in Figure S8b, we observed how the decay in current seem to happen at a slower relative pace. Still, at 1.2 V an XRD shift is observed (Figure S8c).

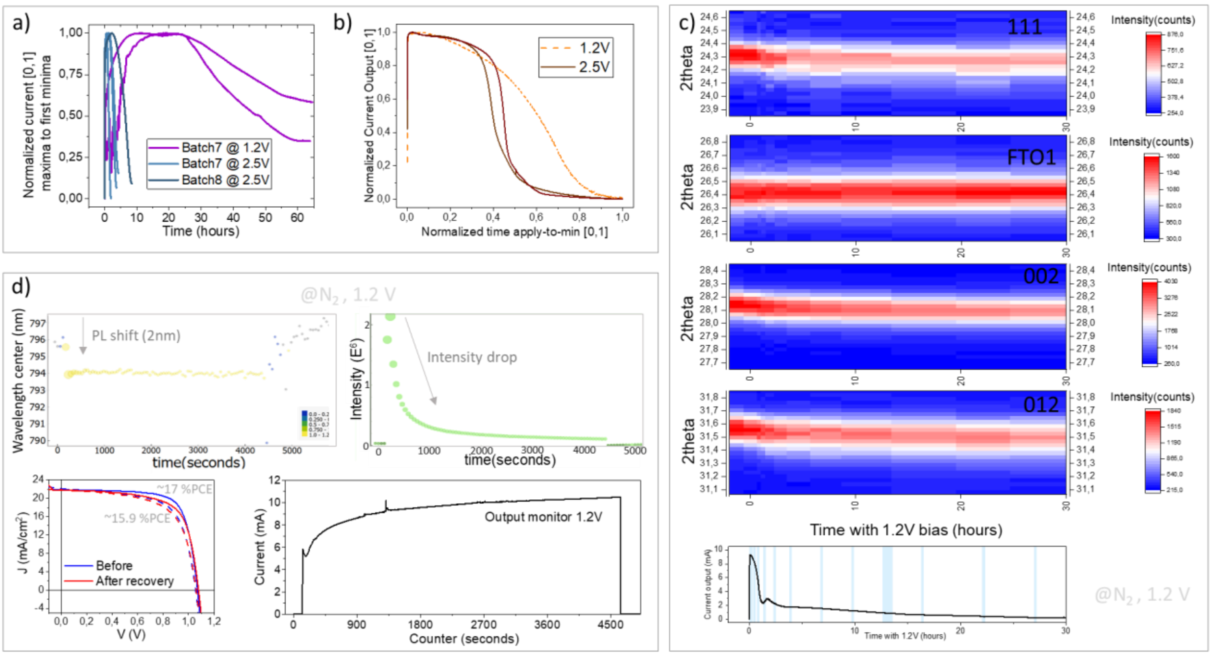

Figure S8. a) Current progression at 2.5 V and 1.2 V normalized intensity [1,0]. b) Intensity normalized as in a) and time normalized from application of bias to first minima to compare the relative slope of the current decay at 1.2 V or 2.5 V. c) XRD shift when applying 1.2 V. d) PL when applying 1.2 V, top: PL center (and intensity) change as fitted by a Gaussian function, bottom: JV comparison before and 1 day after, current output during the experiment.

At lower biases (1.2 V), we observed the same shift in both XRD (Figure S8c) and PL (Figure S8d) analyses, yet of smaller magnitude. The shift as seen on PL (at rising current curve) was at least partially reversible.

S9. XRD two-dimensional correlation map

To detect small changes in the XRD signal, a model-free spectral analysis was performed using two-dimensional correlation map spectroscopy (2DCOS). This analytical method enables the detection of statistically significant changes in a spectrum caused by an external perturbation. Small fluctuations can be identified by analysing the correlation peaks that occur at a specific frequency on the two-dimensional plane. The synchronous contour map, denoted as $\Phi(w_1, w_2)$, represents simultaneous or coincidental changes in scans at two different frequencies, w_1 and w_2, observed over a specific period in response to an external perturbation. Peaks located along diagonal of the map correspond the autocorrelation function, which is similar to the continuous form of statistical variance. These peaks are called auto-peaks. Auto-peaks have always positive values, indicating the degree to which the signal can change in response to an external perturbation. Off-diagonal peaks, also known as cross peaks, indicate simultaneous or coincidental changes in two separate modes detected at coordinates w_1 and w_2. This synchronicity suggests the possibility of a connected or correlated origin for the variations in the signal.[10] Figure S9 shows the synchronous 2DCOS map and autocorrelation plot of REF and MOD samples. Various auto peaks around $2\theta = 24°$, $28°$ and $31.5°$ are observed in synchronous 2DCOS map and in the auto correlation plot associated to the perovskite film. This indicates that the applied voltage (i.e., the external perturbation) induces a significant modification on the peaks of the perovskite film. The rest of the peaks (FTO substrate) did not exhibit a significant

variation in signal, indicating that they are not influenced by the external perturbation. A zoom around the most intense auto-peak shows asymmetrical four-leafed clover pattern. This effect is mainly associated to three coupled effects: upwards shift of the band position, broadening of the peak, and change of the peak intensity of the XRD signal. The upward shift is evident from the asymmetry in the in the auto-plot that show higher intensity at higher angles.

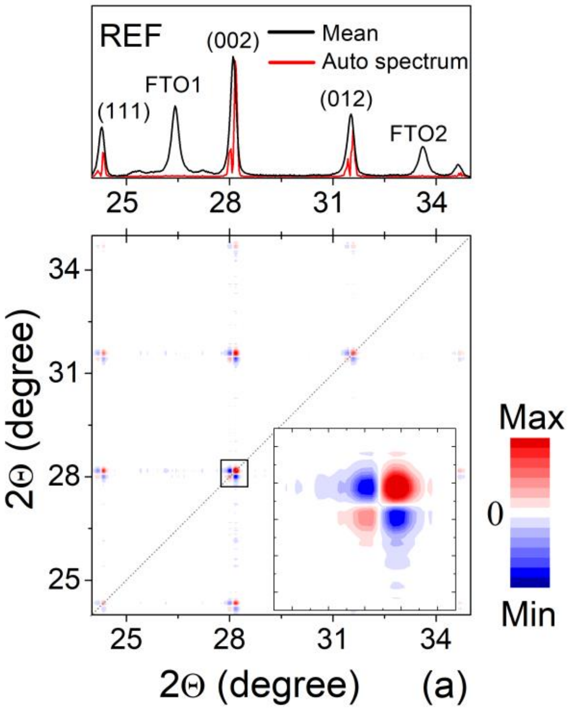

Figure S9. Synchronous two-dimensional correlation map (contour plot) and auto- and mean plot (top) of (a) REF and (b) MOD (with H3pp additive) XRD scans. The dashed line in the contour plot indicates the location of auto correlation plot. (Inset) zoom around the main auto peak.

The results confirmed the significant shift in 2θ angle of the perovskite (111), (002) and (012) directions. Two-dimensional correlation analysis confirmed the progressive modification of the diffraction peaks associated to the HP, indicating crystallinity loss with lack of involvement from other components or phases (Figure S9). The absence of XRD peak shift from the FTO substrate in all samples proved that the XRD changes observed for the HP did not originate from sample alignment drift. We concluded that no other phases than the pure perovskite and non-crystalline phases should be involved in the observed phenomena, and that the conclusion of expansion and loss of crystallinity were supported.

S10. Irreversible decomposition after long stress.

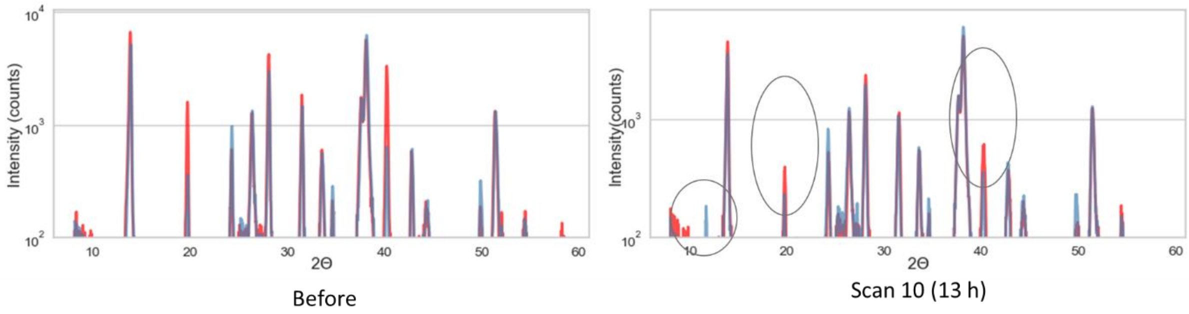

Figure S10.1. Logarithmic scale XRD pattern before and after 13 h of accelerated stress test.

Figure 3e-f in the main text shows a small peak at 2θ = 11.6° towards the end of stress test. Previous studies link this peak at 11.6° to the formation of the photo-inactive hexagonal delta perovskite phase, particularly in compositions based on $FAPbI_3$.[11-15] Additionally, a nearby peak at 2θ = 11.8° has been associated with hydration.[16] However, recent research reveals that this 11.8° peak, observed during hydration, is actually comprised of two peaks at 2θ = 11.65° and 11.8°, with the latter mostly attributed to the hydrated phase of degraded halide perovskite.[17] In several reports of humidity-stressor induced degradation, the peak position tends to be closer to 11.8° rather than 11.6°. In our study, we only observed a single peak at 2θ = 11.6°, suggesting that the material degradation was unlikely due to interaction with atmospheric humidity. A suggested mechanism for the transition from alpha to delta (yellow) phase has been suggested by molecular dynamics (MD) simulations to be the transition from corner-sharing BX_3 octahedra to face-sharing octahedra.[18] Long application of 2.5 V and 0.3 sun (Figure S10.2a) led to a visible amorphization or conversion to non-photoactive delta phase (yellow phase). This phase was visible by eye after removing the cell from the holder (Figure S10.2b), and clearly related to the application of bias.

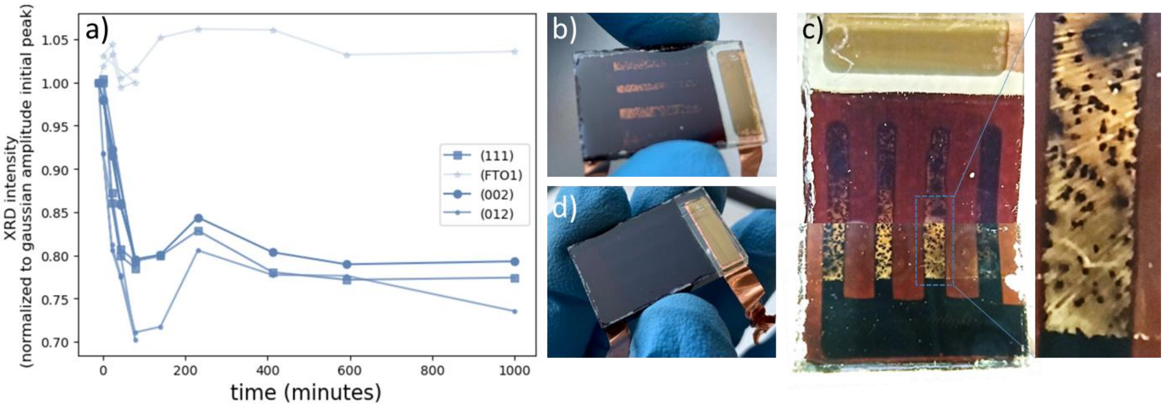

Figure S10.2. a) Normalized amplitude of (111), (002), (012), and FTO XRD peaks measured simultaneously as full PSC device (REF) was subjected to 2.5 V positive bias and 0.3 sun illumination in N_2 atmosphere for ~18 hours. b) Photographs of the tested REF PSC right after testing, c) After 10 days, with additional zoomed-in photo. d) Photograph of MOD PSC after ~18 h of 0.3 sun and 2.5 V external bias applied, internal bias applied only 60 minutes before the cell short circuited.

It is important to note that the electrical bias applied to the finger electrodes is higher/larger than the one applied underneath the thin Au film only. The degradation was most prominent in areas where both light and bias were applied. During short circuit of the device, it seemed that the operation was halted because of the inefficient application of bias in the HP film. The observed changes were still

present 1 year after testing. This change demonstrates and supports the formation of delta phase as a step towards amorphization and the non-reversibility of the observed degradation.

S11. PL over time under bias.

Before applying stress, it was ensured that continuous photoluminescence (PL) measurements did not produce changes in spectra during the time-series. Subsequently, the PL was collected over time with 30 s sweeps from 680-880 nm wavelength detection an integration time of 0.2 s and step size of 2 nm, followed by 30 s of dark rest for the sample before next sweep. After a couple of acquisitions, a constant forward bias was applied and held while collecting the output current.

Each PL spectrum was fitted to a Gaussian function to extract amplitude, center and standard deviation. PL center and amplitude are visualized at a logarithmic time-axis together in Figure S11, with the output current to correlate the evolution of PL spectra with the decay in device performance for a total of around 200 minutes of applied bias. We see that a blue-shift in PL, a bandgap shift of about 20 meV, precedes the onset of current decay.

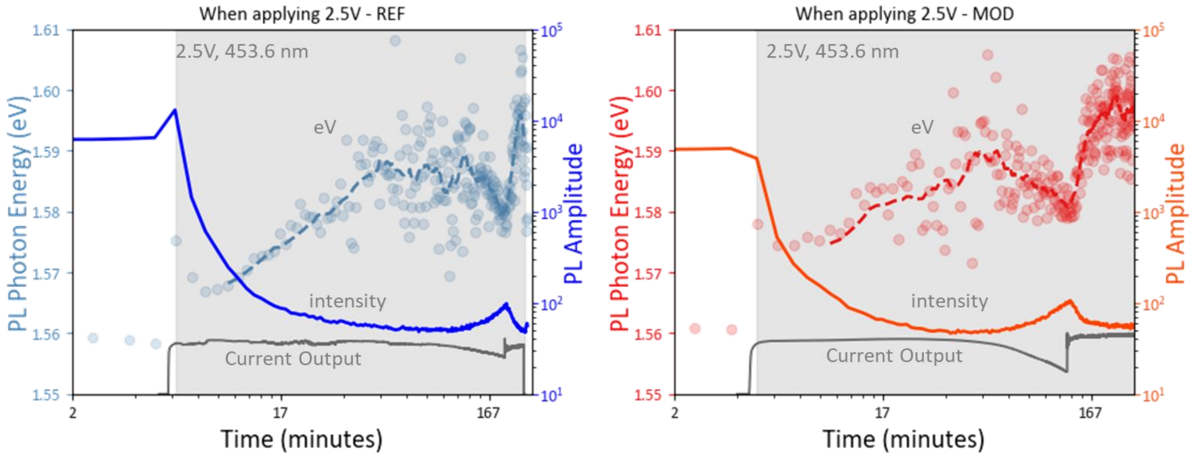

Figure S11. PL center and amplitude as fitted per Gaussian function over time together with the current output progression (2.5 V applied on grey shading). REF (blue) and MOD (red).

The return of the peak center upon the reduction of the current output is supporting of our hypothesis that the current decrease stems from formation of an interfacial barrier. Such a barrier would indeed lead to a decrease in the internal electric field that could to some extent reverse the expansion and accompanied bandgap shift. We also see a slight recovery in the amplitude during this process. In due course during the current output reduction, devices proceeded to short circuit.

We could not exclude that the decay might be more pronounced at these conditions due to the intense interaction with the 453.6 nm laser used to stimulate the photoluminescence.

References

(1) Harrington, G. F.; Santiso, J. Back-to-Basics tutorial: X-ray diffraction of thin films. *Journal of Electroceramics* **2021**, *47* (4), 141-163. DOI: 10.1007/s10832-021-00263-6.
(2) Baumann, F.; Karimipour, M.; R. Raga, S.; Padilla-Pantoja, J.; Caicedo Roque, J. M.; Chávez-Ángel, E.; Alcalá Ibarra, A.; Rémy, P.; Santiso, J.; Lira-Cantu, M. Replication Data for: "Strain in Halide Perovskites and Solar Cell Stability: Accelerated Stress Tests under Bias Voltage"; CORA.Repositori de Dades de Recerca: 2024. Institut Català de Nanociència i Nanotecnologia; https://doi.org/10.34810/data1898

(3) Demsar J, C. T., Erjavec A, Gorup C, Hocevar T, Milutinovic M, Mozina M, Polajnar M, Toplak M, Staric A, Stajdohar M, Umek L, Zagar L, Zbontar J, Zitnik M, Zupan B. Orange: Data Mining Toolbox in Python. *Journal of Machine Learning Research* **2013**, *14* ((Aug)), 2349−2353.
(4) BD Cullity, S. S. *Elements of X-ray diffraction*; Harlow: Pearson Education Limited;, 2014.
(5) Yang, B.; Bogachuk, D.; Suo, J.; Wagner, L.; Kim, H.; Lim, J.; Hinsch, A.; Boschloo, G.; Nazeeruddin, M. K.; Hagfeldt, A. Strain effects on halide perovskite solar cells. *Chemical Society Reviews* **2022**, *51* (17), 7509-7530, 10.1039/D2CS00278G. DOI: 10.1039/D2CS00278G.
(6) Wright, M.; Uddin, A. Organic—inorganic hybrid solar cells: A comparative review. *Solar Energy Materials and Solar Cells* **2012**, *107*, 87-111. DOI: https://doi.org/10.1016/j.solmat.2012.07.006.
(7) Tomohiro, S. M. N. GLASS SUBSTRATE WITH CONDUCTIVE FILM FOR SOLAR CELL. Japan 2012.
(8) Khenkin, M. V.; Katz, E. A.; Abate, A.; Bardizza, G.; Berry, J. J.; Brabec, C.; Brunetti, F.; Bulović, V.; Burlingame, Q.; Di Carlo, A.; et al. Consensus statement for stability assessment and reporting for perovskite photovoltaics based on ISOS procedures. *Nature Energy* **2020**, *5* (1), 35-49. DOI: 10.1038/s41560-019-0529-5.
(9) Prete, M.; Khenkin, M. V.; Glowienka, D.; Patil, B. R.; Lissau, J. S.; Dogan, I.; Hansen, J. L.; Leißner, T.; Fiutowski, J.; Rubahn, H. G.; et al. Bias-Dependent Dynamics of Degradation and Recovery in Perovskite Solar Cells. *ACS Applied Energy Materials* **2021**, *4* (7), 6562-6573. DOI: 10.1021/acsaem.1c00588.
(10) Generalized Two-Dimensional Correlation Spectroscopy in Practice. In *Two-Dimensional Correlation Spectroscopy – Applications in Vibrational and Optical Spectroscopy*, 2004; pp 47-64.
(11) Stoumpos, C. C.; Malliakas, C. D.; Kanatzidis, M. G. Semiconducting Tin and Lead Iodide Perovskites with Organic Cations: Phase Transitions, High Mobilities, and Near-Infrared Photoluminescent Properties. *Inorganic Chemistry* **2013**, *52* (15), 9019-9038. DOI: 10.1021/ic401215x.
(12) Kundu, S.; Kelly, T. L. In situ studies of the degradation mechanisms of perovskite solar cells. *EcoMat* **2020**, *2* (2), e12025. DOI: https://doi.org/10.1002/eom2.12025.
(13) Yi, C.; Luo, J.; Meloni, S.; Boziki, A.; Ashari-Astani, N.; Grätzel, C.; Zakeeruddin, S. M.; Röthlisberger, U.; Grätzel, M. Entropic stabilization of mixed A-cation ABX3 metal halide perovskites for high performance perovskite solar cells. *Energy & Environmental Science* **2016**, *9* (2), 656-662, 10.1039/C5EE03255E. DOI: 10.1039/C5EE03255E.
(14) Jeon, N. J.; Noh, J. H.; Yang, W. S.; Kim, Y. C.; Ryu, S.; Seo, J.; Seok, S. I. Compositional engineering of perovskite materials for high-performance solar cells. *Nature* **2015**, *517* (7535), 476-480. DOI: 10.1038/nature14133.
(15) Sun, Y.; Peng, J.; Chen, Y.; Yao, Y.; Liang, Z. Triple-cation mixed-halide perovskites: towards efficient, annealing-free and air-stable solar cells enabled by Pb(SCN)2 additive. *Scientific Reports* **2017**, *7* (1), 46193. DOI: 10.1038/srep46193.
(16) Ho, K.; Wei, M.; Sargent, E. H.; Walker, G. C. Grain Transformation and Degradation Mechanism of Formamidinium and Cesium Lead Iodide Perovskite under Humidity and Light. *ACS Energy Letters* **2021**, *6* (3), 934-940. DOI: 10.1021/acsenergylett.0c02247.
(17) Yun, J. S.; Kim, J.; Young, T.; Patterson, R. J.; Kim, D.; Seidel, J.; Lim, S.; Green, M. A.; Huang, S.; Ho-Baillie, A. Humidity-Induced Degradation via Grain Boundaries of HC(NH2)2PbI3 Planar Perovskite Solar Cells. *Advanced Functional Materials* **2018**, *28* (11), 1705363. DOI: https://doi.org/10.1002/adfm.201705363.
(18) Lu, H.; Liu, Y.; Ahlawat, P.; Mishra, A.; Tress, W. R.; Eickemeyer, F. T.; Yang, Y.; Fu, F.; Wang, Z.; Avalos, C. E.; et al. Vapor-assisted deposition of highly efficient, stable black-phase FAPbI₃ perovskite solar cells. *Science* **2020**, *370* (6512), eabb8985. DOI: doi:10.1126/science.abb8985.
